# Biallelic editing of a lamprey genome using the CRISPR/Cas9 system

**DOI:** 10.1038/srep23496

**Published:** 2016-03-23

**Authors:** Yao Zu, Xushuai Zhang, Jianfeng Ren, Xuehong Dong, Zhe Zhu, Liang Jia, Qinghua Zhang, Weiming Li

**Affiliations:** 1 College of Fisheries and Life Science, Shanghai Ocean University, Shanghai 201306, China; 2 Department of Fisheries and Wildlife, Michigan State University, East Lansing, MI 48824, USA

## Abstract

Lampreys are extant representatives of agnathans. Descriptions of lamprey development, physiology and genome have provided critical insights into early evolution of vertebrate traits. However, efficient means for genetic manipulation in agnathan species have not been developed, hindering functional studies of genes in these important Evo-Devo models. Here, we report a CRISPR/Cas system optimized for lamprey genomes and use it to disrupt genomic loci in the Northeast Chinese lamprey (*Lethenteron morii*) with efficiencies ranging between 84~99%. The frequencies of indels observed in the target loci of *golden* (*gol*), *kctd10, wee1, soxe2*, and *wnt7b*, estimated from direct sequencing of genomic DNA samples of injected lamprey larvae, were 68/69, 47/56, 38/39, 36/37 and 36/42, respectively. These indels often occurred in both alleles. In the CRISPR/Cas9 treatment for *gol* or *kctd10*, 38.6% or 85.3% of the targeted larvae had the respective recessive null-like phenotypes, further confirming the disruption of both loci. The *kctd10* gRNA, designed against an essential functional region of Kctd10, resulted in null-like phenotypes and in-frame mutations in alleles. We suggest that the CRISPR/Cas-based approach has the potential for efficient genetic perturbation in organisms less amenable to germ line transmission based approaches.

Lampreys and hagfishes are sole surviving lineages of jawless vertebrates, or agnathans. As an out-group to the gnathostomes (vertebrates with hinged jaws), the development and evolution of agnathans have been examined extensively. These studies have provided important insights into the origin and early radiation of the vertebrate linage^1^,^2^,^3^. In particular, studies in lampreys have helped reveal possible developmental mechanisms of the craniate, and the origin of the features unique to vertebrate animals, such as the neural crest, the true brain, the pharyngeal skeleton, the jaw, the vertebra and the appendage^2^,^4^. In addition, lampreys have been used extensively as biomedical models for elucidation of mechanisms that govern adaptive immunity, spinal cord regeneration, locomotion, and neuronal degeneration^5^,^6^.

With a few exceptions, such as elucidation of particular gene functions through morpholino-knockdowns and RNA interference technology^7^,^8^,^9^, studies of lamprey development and gene function have remained largely descriptive. To define the functions of essential genes in an organism, both genome data and a means to edit the genome are indispensable. The draft genomes of two lamprey species, the sea lamprey (*Petromyzon marinus*) and Japanese lamprey (*Lethenteron japonicum*), have become available^10^,^11^. However, techniques for genetic mutagenesis in lamprey species have not been established. Established techniques that generate mutations through germ line transmission are difficult to implement in lamprey species, which develop through a lengthy (up to 20 years to reach sexual maturation) and complicated life cycle^3^,^12^,^13^,^14^. Not surprisingly, culture of a lamprey (or any agnathan) species through a complete life cycle in captivity has not been reported. Furthermore, lampreys are semelparous species; sexually mature adults spawn once and expire, making it exceedingly difficult to obtain sufficient F_1_ and F_2_ individuals even if one can cultivate lamprey to maturation. We reasoned that a method that efficiently disrupts both alleles of genomic loci in the founder (F_0_) generation would be amenable for studies of gene function in lamprey species. Furthermore, many species that are uniquely advantageous for addressing certain biological questions but less tractable genetically through germ line transmission based approaches should also become more useful as model systems when genome editing at the (F_0_) generation becomes available.

Recently, a series of designer nucleases targeting specific endogenous DNA sequences has been developed^15^,^16^. Among these systems, the CRISPR/Cas (clustered regularly interspaced short palindromic repeats/CRISPR-associated proteins) system emerged as a method of choice for its simplicity and efficiency in introducing mutations of target genes in a variety of model organisms^17^,^18^,^19^,^20^. Furthermore, CRISPR/Cas9 systems with high cleavage activities have been shown to cut both alleles of a target gene efficiently in embryonic stem cells as well as in organisms including mouse, pig and zebrafish^21^,^22^,^23^,^24^.

Here we report the development of a CRISPR/Cas9 system that targets genomic loci in lamprey larvae with high efficiencies. Since the CRISPR/Cas9 system relies on an RNA-guided nuclease system derived from prokaryotes, the Cas9 nuclease activity may be enhanced if its cDNA is optimized according to the codon usage in the target eukaryote genome. We applied the optimized CRISPR/Cas9 system in five loci, *wee1, soxe2, kctd10, wnt7b* and *golden* (*gol or slc24a5*). These genes were selected because they have confirmed functions in vertebrate development or give convenient and consistent readouts of phenotypes, or both^7^,^8^,^25^,^26^. In particular, the *gol* encodes the sodium/calcium exchanger Slc24a5 that affects pigmentation in zebrafish and human. A mutation of *gol* in zebrafish, *gol*^*b1*^, results in hypopigmentation of skin melanophores and retinal pigment epithelia^27^. The loss of function for *gol* is recessive, and therefore the retinal color pattern is a convenient readout for testing the efficiency of biallelic loci disruption. *kctd10* encodes the potassium channel tetramerization domain-containing 10. In zebrafish, mutation of *kctd10* causes malformation of the atrioventricular canal, resulting in a stretched and string-like heart^28^. The loss-of-function of *kctd10* is recessive, and the phenotype of the mutant is consistently recognizable. In the Northeast Chinese lamprey (*Lethenteron morii*), injection of the *Cas9* mRNA optimized for lamprey codon usage along with the relevant gRNAs into one-cell stage embryos disrupted both alleles of each of the five endogenous genes in F_0_. In the two loci, *gol* and *kctd10*, that give clear phenotype readouts in the larval stage, the null-like phenotypes were observed in 38.6% and 85.3% of the founders, respectively. We conclude that the CRISPR/Cas9-based technique is an efficient means to disrupt both alleles of endogenous genes in lamprey.

## Results

### CRISPR/Cas9 system mediated gene targeting in lamprey

We examined three Cas9 proteins for their efficiency in altering lamprey alleles: 1) ZCas9, the *Streptococcus pyogenes* Cas9 optimized for zebrafish codon usage with an SV40 NLS at the 5′ end and a nucleoplasmin NLS at the 3′ end^29^; 2) LCas9-1 and 3) LCas9-2, both of which were optimized according to the codon usage determined from 15,790 coding sequences of the sea lamprey draft genome (Fig. S1, Table S1). LCas9-1 and LCas9-2 differed slightly in their amino acid residue sequences. The codon optimized LCas9-1 and -2 were fused to NLS at both 5′ and 3′ ends (Fig. S1). The three *Cas9* cDNAs were ligated separately into a *pxT7* expression vector, and transcribed individually into *Cas9* mRNAs. The guide RNAs (gRNAs) against the loci of the five targeted endogenous genes of lamprey (Fig. S2 and Table S2) were designed and synthesized. The according gRNA for each examined gene was injected, along with each of the three *Cas9* mRNAs simultaneously, respectively, into lamprey embryos at the one-cell stage, resulting in a total of 15 combinations of gRNA and *Cas9* mRNA.

**Figure 1:**
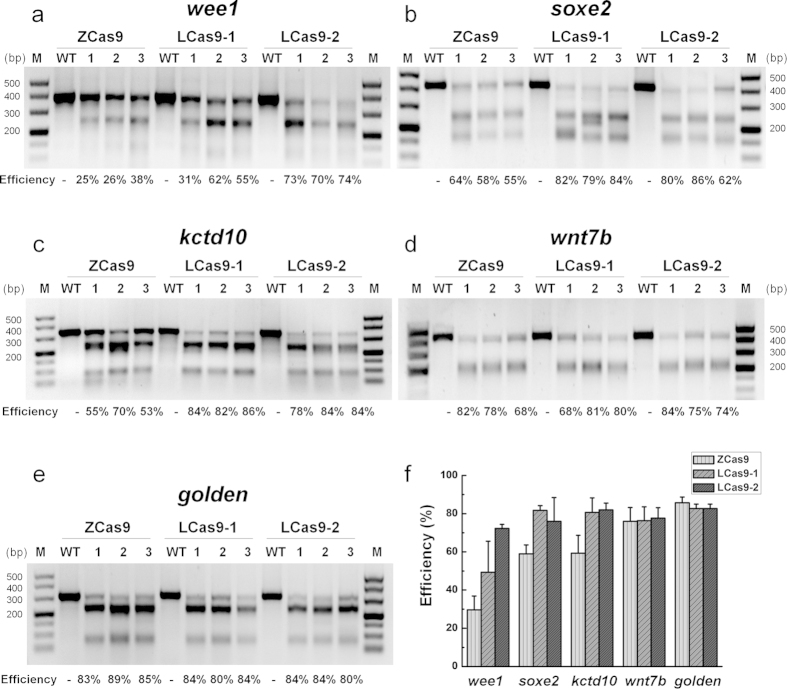
The CRISPR/Cas9 system induced mutagenesis in five endogenous genes of lamprey. *Cas9* mRNA and gRNA against each of the five endogenous genes were injected separately into lamprey embryos at the one-cell stage. The control embryos (WT) were wild type. T7EI assays showed the mutagenesis rates in three groups of pooled injected embryos for each target site, and each group contained five randomly selected individual. For every endogenous gene and for every type of Cas9, the rates of mutagenesis ranged from 25% to 89%.

The T7 Endonuclease I (T7EI) assay^30^ revealed robust mutagenesis at all five loci targeted by the CRISPR/Cas9 systems (Fig. 1). All tested Cas9s cleaved the target regions of the lamprey genome in the presence of the appropriate gRNA. In most cases, the Cas9s optimized for the lamprey genome appeared to be more efficient (Fig. 1f). Approximately 60% to over 80% of the PCR products derived from the target regions of these five genes were cleaved by T7EI when LCas9-2 was used (Fig. 1). These data demonstrate that the optimized CRISPR/Cas9 systems targeted the lamprey genomic loci effectively.

### Treatment of Cas9 and *gol* gRNA induced null-like phenotypes in F_0_

To further evaluate the efficiency of the optimized CRISPR/Cas9 systems in inducing biallelic disruptions of endogenous genes in lamprey, we targeted *gol* loci in *L. morii*. Analyses through sequence alignment indicated that Slc24a5 is conserved within the vertebrate clade. The conservation of amino acid residues between lamprey and human, mouse or zebrafish are at 54%, 55% and 56% identity, respectively (Fig. S3), suggesting that Slc24a5 may have similar functions in these animals.

**Figure 2:**
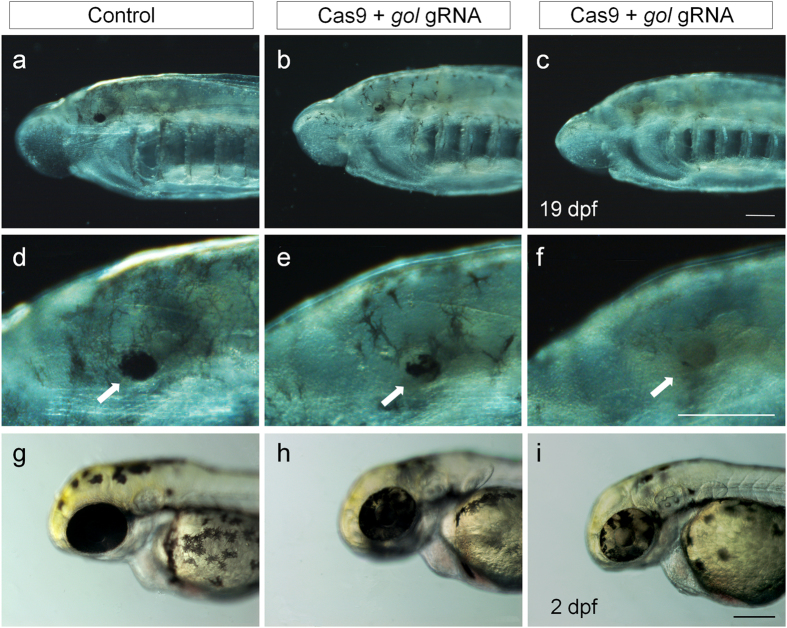
Retina pigmentation in lamprey and zebrafish developed from Cas9 and *gol* gRNA injected embryos. (**a**–**f**) Lateral views of control (**a**,**d**) and *gol* gRNA injected larvae (**b**,**c**,**e**,**f**) on 19 dpf (days post fertilization). Phenotypes shown include the wild-type (arrow in (**d**)), mosaic hypopigmentation (arrow in (**e**)) and no observable pigment (arrow in (**f**)) in the retina. Zebrafish embryos injected with *Cas9* and the appropriate *gol* gRNA on 2 dpf also showed mosaic pigmentation (**h**,**i**) in the retinas. Scale bars: 0.2 mm.

To target the loci of *gol, Cas9* mRNAs were injected into one-cell stage lamprey embryos along with *gol* gRNA (Fig. S2). The *gol* gRNA was designed to target a DNA segment found in the coding region of lamprey *gol* but absent in the human, mouse or zebrafish orthologs (Fig. S3). The retina of *L. morii* larvae was examined for pigmentation on day 19 after the injection (Table 1). The injection largely affected the rate of null-like phenotypes (χ^2^ = 170.24, *p* < 0.0001). Among the larvae (n = 132) injected with LCas9-1 and *gol* gRNA, 67.4% showed hypopigmentation, of which 38 larvae (28.8% of the injected larvae) had mosaic hypopigmentation (Fig. 2b,e, and Fig. S4d–f). A total of 51 larvae (38.6% of the injected larvae) had no observable pigment in their retinas, indicating that biallelic disruption of *gol* occurred in the majority of the cells in these individuals (Fig. 2c,f, and Fig. S4g–S4i). LCas9-1 and LCas9-2 differed in their effects on pigmentation patterns (χ^2^ = 10.01, *p* = 0.007), with LCas9-1 inducing a higher rate of the null-phenotype and LCas9-2 inducing a higher rate of mosaic pigmentation in the retina (Table 1).

As a positive control and to compare the efficiency of allelic disruption, we targeted the *gol* loci in zebrafish using ZCas9^29^ and an appropriate gRNA^21^. The injected zebrafish larvae showed the three pigmentation phenotypes similar to those obtained in lamprey larvae (Fig. 2g–i), confirming that the function of *gol* is highly conserved in vertebrates. Notably, the percentage of the *gol* null-like phenotype was drastically lower than the mosaic pigment phenotype in zebrafish compared to lamprey (Table S3). This discrepancy is most likely because CRISPR/Cas9 based biallelic disruption of *gol* in founders was more efficient in the lamprey than in the zebrafish.

### Treatment of Cas9 disrupted both alleles of *gol* in virtually all cells

We sequenced the target region of the genome from injected animals to examine the exact alteration induced by the CRISPR/Cas9 systems at the lamprey *gol* locus. Genomic DNA extracted from five founder individuals (two with no pigment, two with mosaic pigment and one with normal pigment) were examined. Twenty independent colonies derived from each DNA sample were sequenced (Table 2 and Fig. S5). The genomic DNA sequences showed that both alleles of *gol* in most cells were disrupted in the null-like phenotype larvae. In one larva with no observable retinal pigments (N2), all 18 valid sequences showed indels as compared to those obtained from a wild-type lamprey, indicating that both alleles of *gol* were targeted (Table 2 and Fig. S5a). Seven different types of indels were found in this individual, indicating that the CRISPR/Cas9 enabled mutation occurred independently in individual cells. Most indels (14 out of 18) in this larva caused a frame shift or large fragment deletion, resulting in a null-allele. These results demonstrate that both alleles of *gol* in most cells of the larvae with the null-like phenotype were disrupted.

To our surprise, the genomic DNA sequence of injected larvae that did not display the null-like phenotype only contained mutated alleles of *gol*. The 34 valid sequences from the two larvae with mosaic pigmentation all contained mutated *gol* alleles (Table 2 and Fig. S5b). However, many deletions (10 out of 18 in one larva and 7 out of 16 in the other) caused in-frame mutations, and conserved amino acid residues were not affected in most of these deletions. Even the injected larva that had normal retinal pigmentation contained indels in all 17 valid sequences, although the majority of the mutations (13 out of 17) were in-frame deletions (Table 2 and Fig. S5c).

Collectively, among the 86 sequences obtained from the five injected lamprey larvae representing three different phenotypes, only one sequence was the wild-type. Clearly, injection of LCas9-1 and *gol* gRNA into lamprey embryos at the one-cell stage induced mutations in almost all the cells of the larvae, and virtually all disruptions of the genomic locus were biallelic. These results demonstrate that the optimized CRISPR/Cas9 system modified both *gol* alleles at an efficiency approaching 99% in F_0_.

### Treatment of Cas9 and *kctd10* gRNA induced null-like phenotypes in F_0_

We next evaluated the utility of the optimized CRISPR/Cas9 system in targeting the biallelic loci of genes essential for animal development. We chose *kctd10* that plays an essential role in heart morphogenesis^28^. The lamprey Kctd10 protein shares 85~87% amino acid sequence identity with its zebrafish, mouse and human orthologs (Fig. S6). We predicted that Kctd10 may have a similar role in cardiac development in these organisms.

**Figure 3:**
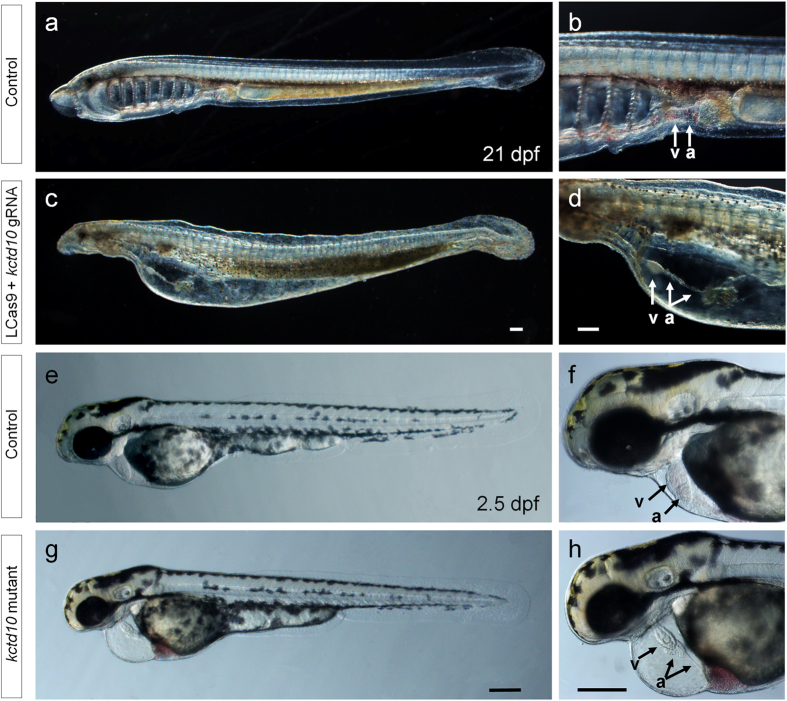
Heart malformations in lamprey larvae and zebrafish developed from *Cas9* and *kctd10* gRNA injected embryos. (**a**–**d**) Lateral views of control (**a**,**b**) and *kctd10* and gRNA injected lamprey larvae (**c**,**d**) on 21 dpf (days post fertilization). The control larvae have normal heart formation (**a**) and the treatment larvae have severe pericardial edema (**c**). (**b**,**d)** enlarged views of **a** and **c**, respectively, provide a comparison that shows the marked heart malformations in the injected larvae (**d**). (**d**) the atrium (**a**) stretches to a string-like structure and no blood was observed in either atrium or ventricle (v). Zebrafish larvae of *kctd10* mutant had pericardial edema (**g**) and heart malformations (**h**) as compared to the wild-type larvae (**e**,**f**) on 2.5 dpf. In the mutants, part of the atrium stretches to a string-like structure and no blood was observed in either atrium or ventricle (**h**). Scale bars: 0.2 mm.

Lamprey embryos at the one-cell stage were injected with *Cas9* mRNA and *kctd10* gRNA, and their morphology observed during development. The *kctd10* gRNA was designed to target a conserved region of the coding sequence (Fig. S6). The Cas9 treatments significantly affected the rate of heart malformation (χ^2^ = 169.61, *p* < 0.0001). LCas9-1 induced a higher rate of heart malformations than LCas9-2 (χ^2^ = 17.02, *p* < 0.0001) and ZCas9 (χ^2^ = 5.28, *p* = 0.022). Twenty one days after injection of larvae with LCas9-1 and *kctd10* gRNA, 99 out of 116 (85.3%) showed severe pericardial edema (Table 3, Fig. 3c, and Fig. S7c,S7d). The malformed hearts had a stretched, string-like morphology and beat weakly (Fig. 3d). The zebrafish *kctd10* mutant, previously identified from a spontaneous recessive mutation^28^, also has a string-like heart morphology and weakened heart contractility (Fig. 3g,h). Clearly, treatment of optimized Cas9 and *kctd10* gRNA in lamprey resulted in a phenotype similar to that of zebrafish *kctd10* mutants. These results suggest that both alleles of *kctd10* in the injected lamprey larvae were disrupted, and the function of Kctd10 is conserved between lamprey and zebrafish.

### The Cas9 system disrupted both alleles of *kctd10* in lamprey

We genotyped the *kctd10* loci in six LCas9-1 injected lamprey larvae (Table 4 and Fig. S8). For the three larvae that displayed heart malformations, we obtained 20, 19 and 17 valid sequencing results, respectively. Among the *kctd10* sequences, 16, 18 and 13, respectively, contained indels (Fig. S8). Some of which caused a frame shift and others (>50%) caused deletions in amino acid residues that are conserved between human, mouse, zebrafish and lamprey. Overall, the frequency of the wild-type alleles of *kctd10* was very low (4/20, 1/19 and 4/17), indicating that most cells of these individuals contained only mutated alleles. In other words, both alleles of *kctd10* were targeted in most cells of the larvae with heart malformations. The three injected larvae with no overt heart malformations, had higher ratios of wild-type sequences (8/18, 9/20 and 13/19; Table 4 and Fig. S8b). Taken together, these results reveal that LCas9-1 efficiently mutated both alleles of *kctd10*, when introduced into lamprey embryos at the one-cell stage, leading to null-like phenotypes in the founder generation.

### Cas9 targeting in the loci of *wee1, soxe2* and *wnt7b*

We also confirmed by sequencing biallelic disruption in the genomic loci of *wee1, soxe2* and *wnt7b*, in the larvae injected with the *Cas9* mRNA and appropriate gRNA. Although mutated alleles of each gene were detected by the T7EI assay (Fig. 1), no apparent mutated phenotypes were observed in the larval stage. We determined if the Cas9 treatment disrupted both alleles of these loci in the target regions (Fig. S9) using the strategy outlined for the *gol* and *kctd10* loci genotyping.

Our sequencing results showed that LCas9-1 disrupted both alleles of *wee1, soxe2* or *wnt7b* at very high levels of efficiency. For the target region of *wee1*, 39 of the 40 valid sequences contained mutations, for the *soxe2* target region, 36 of the 37 sequences contained indels and for the *wnt7b* target region, 36 of the 42 sequences contained indels. It was evident that indels occurred in both alleles of all three genes. These results further confirmed that injection of *Cas9* mRNA together with the appropriate gRNA efficiently target both alleles of endogenous genes in lamprey F_0_.

## Discussion

Lamprey species are used extensively in development and evolution studies^3^. However, these species are not readily amenable to either forward genetics or reverse genetics, the traditional approaches that rely on the mutations recovered and maintained through germ line transmission. In these approaches, mutations are introduced into the F_0_, or founder organism. Usually, the F_0_ is mosaic for the mutation, and is bred with the wild-type to generate the F_1_, some of which are heterozygous for the mutation. In reverse genetics, it is the cross between the F_1_ heterozygotes that results in the homozygote mutant. In forward genetics, the heterozygotes of the same mutation are obtained in the F_2_^31^,^32^. However, it is technically challenging and costly to culture lamprey embryos to adult stages under laboratory conditions because lamprey species develop through a lengthy and complex life cycle that demands dramatic habitat changes to support the transition between life stages^12^. Therefore, it is not yet practical to transmit introduced mutations from F_0_ to F_1_ in this group of animals. Nonetheless, manipulation of fertilized lamprey eggs is possible as the chorions are resistant to rupture induced by surface-tension and like zebrafish and *Xenopus* embryos, the one-cell stage embryo can be injected in dry dishes^33^. The one-cell stage during lamprey embryonic development lasts for several hours, providing a very large injection window and likely increasing the cleavage efficiency of the CRISPR/Cas9 system. In the present study, we disrupted both alleles of all five examined endogenous loci in a lamprey genome by introducing the CRISPR/Cas9 system at the one-cell stage. The efficiency of biallelic mutagenesis ranged from 84% to 99%, which is comparable to that in zebrafish and cynomolgus monkey^21^,^34^, and higher than that in pig^35^.

For the two endogenous genes examined in detail, *gol* and *kctd10*, their disruption led to marked and expected phenotypes in F_0_. When the *gol* loci was targeted, nearly 40% of the injected larvae lacked pigment in their retina, a phenotype of the null mutation, which is similar to that in zebrafish^21^,^36^. Moreover, targeting the *kctd10* loci resulted in an even higher ratio of the null-like phenotype, and up to 85% of injected larvae had a heart malformation that was virtually identical to those observed in *kctd10* deficient zebrafish^28^. At the null-allele rates demonstrated for these two loci, the LCas9-1 based procedure is sufficiently efficient and effective in editing the lamprey genome, with practical utilities in examination of gene function in the founder generation. Although the efficiencies of biallelic disruption of the loci of *wee1, soxe2* and *wnt7b* were also high, no marked disorders were observed in the larvae. A plausible explanation for the lack of the null phenotype is functional compensation by other genes. Another possibility is that the CRISPR/Cas9 and morpholino treatments exert different effects on the target loci, as has previously been shown in zebrafish embryos^37^.

A notable dichotomy in the outcomes of CRISPR/Cas9 treatment of lamprey embryos was the higher rate of frame-shifting indels in the *gol* locus than in the *kctd10* and the dramatically higher null-phenotype rate from targeting *kctd10* compared to *gol*. The *gol* locus, targeted by our optimized CRISPR/Cas9 system, led to a null-phenotype rate of approximately 40%, which is consistent with the theoretical value. In theory, the ratio of the indels that cause reading frame shift over the total indels is 2/3. Hence the probability for both alleles of a targeted locus to carry a frame shift mutation is 4/9, suggesting that 5/9 cells from an injected organism should have the wild-type or in-frame mutation alleles. To overcome this issue, we designed the *kctd10* gRNA to target an essential functional domain that encodes highly conserved amino acid residues. Substitution or deletion of such amino acid residues often lead to a null mutation^38^. As expected, 85% of the larvae injected with Cas9 and gRNA against *kctd10* showed the null-phenotype and had heart disorders, and more than 50% of the null alleles contained in-frame mutations. We suggest to improve efficiency of the CRISPR/Cas9 system to study gene function in the F_0_, highly conserved domains should be targeted rather than randomly selected regions.

The gRNA guided targeting of Cas9 in genomic loci appears to extend beyond the very lengthy one-cell stage of lamprey embryos. This is evident from the seven types of indels documented in genomic DNA of the target region of *gol* in injected individuals. If the targeting process happened only at the one-cell stage, as many as two types of indels for each targeted loci would be expected in the injected individual. We also noticed that the biallelic disruption of endogenous genes by CRISPR/Cas9 systems was more efficient in F_0_ lamprey than in F_0_ zebrafish. This could be explained by the very different durations of early embryonic development in these two animals. In lamprey embryos, the one-cell stage lasts for 5–6 hours, and each subsequent cleavage lasts for about 1 h at 18 °C (Fig. S10). In comparison, the one-cell stage in zebrafish embryos lasts for 45 minutes, and the subsequent cleavage each lasts for 15 minutes at 28.5 °C^39^. In future studies, it would be interesting to examine the functional dynamics of *Cas9* mRNA and gRNA molecules in lamprey and zebrafish embryos through the first few stages, and infer why the Cas9 treatment results in a higher efficiency of biallelic disruptions in the lamprey founders.

In summary, we optimized a CRISPR/Cas9 system that disrupted both alleles of all five examined endogenous genes in a lamprey genome. The biallelic disruption is efficient enough to generate sufficient numbers of the null-phenotype and null-mutation in F_0_ for genetically tractable studies of gene functions. This method will likely enable functional studies of the genes important for the development of lamprey and for understanding early evolution of vertebrate traits. Similar approaches, with straightforward optimizations, may become available to define gene function in other organisms for which F_1_ and F_2_ generations are difficult to generate in laboratory conditions.

## Material and Methods

### Animals and embryos

The Northeast Chinese lampreys were obtained from the Shuifen reservoir, a tributary of Yalu River in Dandong city, Liaoning province, China. Sexually mature lamprey were transported to Shanghai Ocean University campus (Lingang, Shanghai, China), and maintained at 8 °C in tanks with aerated tap water. The eggs were striped from ovulatory females into a dish containing 300 ml autoclaved double distilled water, and the sperm from a spermiating male were expressed onto the eggs for *in vitro* fertilization^40^. As described previously^28^, zebrafish were raised and maintained at 28.5 °C in a re-circulating system with continuously filters. The re-circulated water was treated with UV light and aerated. Breeding was carried out in-tank to obtain the embryos, and the embryos were raised in the incubator at 28.5 °C. All handling of fishes was carried out in accordance with the guidelines on the care and use of animals for scientific purposes set up by the Institutional Animal Care and Use Committee (IACUC) of the Shanghai Ocean University (SHOU), Shanghai, China. This research was approved by the IACUC of SHOU.

### *Cas9* plasmids

The LCas9-1 coding sequence was from *Streptococcus pyoge*nes MGAS1882 (YP_005388840)^21^. The LCas9-2 coding sequences were designed according to Liu *et al*.^29^. Both Cas9 coding sequences were codon-optimized and ordered from Sangon Biotech. The codon usage frequency of sea lamprey is listed in the Table S1. A Kozak consensus was put at the translational start and nuclear localization signal (NLS) was fused at both ends. The full length of codon-optimized *Cas9* sequences with double NLS was cloned into the *pXT7* vector containing T7 promoter and globin UTR. See Fig. S1 for the full sequences of the codon-optimized *LCas9*s.

### RNA Synthesis

All three *Cas9* plasmids were linearlized by *Xba*I. Capped mRNAs of *Cas9s* were synthesized *in vitro* by mMESSAGE mMACHINE T7 ULTRA kit (Ambion), and purified using RNeasy Mini Kit (Qiagen). Double strand cDNA for specific gRNA was amplified by PCR (NEB) using corresponding primers (**target site** specific forward primer 5′-TAATACGACTCACTATA**NNNNNNNNNNNNNNNNNNNN**GTTTTAGAGCTAGAAATAGC-3′; common reverse primer 5′-AAAAAAAGCACCGACTCGGTGCCAC-3′). The template sequence is GTTTTAGAGCTAGAAATAGCAAGTTAAAATAAGGCTAGTCCGTTATCAACT TGAAAAAGTGGCACCGAGTCGGTGCTTTTTTT^41^. Each target sequence contains the GG at the beginning, which is required for T7 RNA polymerase starting. After gel extraction of the PCR product, gRNA was synthesized using MAXIscript T7 kit (Ambion), and digested with DNase I (Ambion) for 15 min to remove DNA. The gRNA was purified using mirVana™ miRNA Isolation Kit (Ambion).

### Microinjection

The microinjection of the Northeast Chinese lamprey embryos followed the protocol of Nikitina^33^ with modification. Briefly, the fertilized eggs (one-cell stage) were washed with 18 °C autoclaved double distillted water for several times, placed on a microinjection dish with a nylon mesh at 18 °C, and each embryo injected with a mixture of the *Cas9* mRNA (400 pg) and gRNA (80 pg). Over 200 embryos were injected for each combination of *Cas9* mRNA and gRNA.

The microinjection of zebrafish embryos was performed at the one-cell stage as described previously^42^. Approximately 400 pg mRNA encoding Cas9 and 80 pg gRNA were injected into each embryo.

### T7EI assay

Five days after injection of each combination of gRNA and *Cas9* mRNA, genomic DNA was isolated from three groups of pooled injected larvae, and each group contained five randomly selected individuals. The target region was amplified with PCR using the according primer pairs (for details, see Table S2). The resulted products were denatured and annealed, and treated with T7 Endonuclease I (NEB) for 45 min at 37 °C. The digested PCR products were examined by agarose gel (2%) electrophoresis.

### Sequence analysis

The genomic DNA was isolated from the injected individual, and the target regions were amplified from the genomic DNA using the according specific PCR primers. Each product was ligated into the *pMD19-T* TA cloning vector (Takara) and transformed into DH5α competent cells (Tiangen). For each amplicon, approximately 20 independent colonies were sequenced by Dye terminator sequencing.

### Imaging

Embryos were treated with 0.02% tricaine (3-amino benzoic acid ethyl ester), mounted in 3% methyl-cellulose, and visualized either under a Stereoscopic Microscopes (Zeiss, Discovery. V20) or a Fluorescence Microscope (Zeiss, Axioimager. M2).

## Additional Information

**How to cite this article**: Zu, Y. *et al*. Biallelic editing of a lamprey genome using the CRISPR/Cas9 system. *Sci. Rep.* **6**, 23496; doi: 10.1038/srep23496 (2016).

## Supplementary Material

Supplementary Information

## Figures and Tables

**Table 1:** Retina pigmentation of lamprey larvae from Cas9 and *gol* gRNA treatment.

Treatment	Total Number of larvae	Normal pigment	No pigment	Mosaic pigment
Control	116	116	0 (0%)	0 (0%)
LCas9-1 + *gol* gRNA	132	43	51 (38.6%)	38 (28.8%)
LCas9-2 + *gol* gRNA	109	33	25 (22.9%)	51 (46.8%)
ZCas9 + *gol* gRNA	90	32	25 (27.7%)	33 (36.7%)

Each Cas9 induced hypopigmentation in comparison to control (p < 0.0001 for each Cas9). LCas9-1 and LCas9-2 differed in inducing the pigmentation patterns (χ^2^ = 10.01, p = 0.007), with LCas9-1 inducing a higher rate of null-phenotypes whereas LCas9-2 inducing a higher rate of mosaic pigmentation in the retina.

**Table 2:** Different alleles of *gol* in the injected larvae.

Phenotype	Sample ID	Total	Wild type	In-frame indels	Frame-shift indels	Null mutation (%)
WT-like	W5	17	0	13	4	23.5
Mosaic	M2	18	0	10	8	44.4
	M5	16	0	7	9	56.3
Null-like	N2	18	0	5	13	72.2
	N5	17	1	4	12	70.6

Total, total number of valid sequence; WT-like, wild type like.

**Table 3:** Larval heart malformations from Cas9 and *kctd10* gRNA treatment.

Treatment	Total Number of larvae	Normal larvae	Larvae with heart disorders	Mutant (%)
Control	114	111	3	2.6
LCas9-1 + *kctd10* gRNA	116	17	99	85.3
LCas9-2 + *kctd10* gRNA	44	20	24	54.5
ZCas9 + *kctd10* gRNA	54	16	38	70.4

Overall, treatment of Cas9 affected the phenotype (χ^2^ = 169.61, *p* < 0.0001). Treatment by each one of the three Cas9s induced more malformations than the vehicle treatment (*p* < 0.0001 for each comparison). LCas9-1 induced a higher rate of malformation in the heart than LCas9-2 (χ^2^ = 17.02, *p* < 0.0001) and ZCas9 (χ^2^ = 5.28, *p* = 0.022). LCas9-2 did not differ from ZCas9 in the number of induced malformations (χ^2^ = 2.613, *p* = 0.10).

**Table 4:** Different *kctd10* alleles in the injected larvae.

Phenotype	Sample ID	Total	Wild type	In-frame indels		Frame-shift indels	Null mutation (%)
				Conserved aa not affected	Conserved aa affected		
WT-like	W1	18	8	0	7	3	55.6
	W2	20	9	0	5	6	55.0
	W3	19	13	0	4	2	31.6
Heart disorder	H1	20	4	0	9	7	80.0
	H2	19	1	0	10	8	94.7
	H3	17	4	0	8	5	76.5

Total, the total number of valid sequence analysed; WT-like, wild type-like; aa, amino acid.
