# Supplementary material for: Biallelic editing of a lamprey genome using the CRISPR/Cas9 system
Source: Sci Rep. 2016 Mar 23;6:23496. doi: 10.1038/srep23496 (PMC4804306; doi:10.1038/srep23496)
Supplement: Supplementary Information [file srep23496-s1.pdf]

Supplementary Information

Biallelic editing of a lamprey genome using the CRISPR/Cas9 system

Yao Zu[1,+], Xushuai Zhang[1,+], Jianfeng Ren[1], Xuehong Dong[1], Zhe Zhu[1], Liang Jia[1], Qinghua Zhang[1], Weiming Li[1,2,]*

[1]College of Fisheries and Life Science, Shanghai Ocean University, Shanghai 201306, China
[2] Michigan State University, Department of Fisheries and Wildlife, East Lansing, MI 48824, USA

Figure S1.

>LCas9-1

Globin 5' UTR   NLS

taatacgactcactatagggagagcttgttctttttgcagaagctcagaataaacgctcaactttggcagatctcgagccacc atggcttctccccctaagaag

Lamprey codon optimized Cas9-1

aagcgaaaggtgggctcc**ATGGACAAGAAATACTCAATTGGGCTGGATATTGGAACTAACTCAGTCGGATGGGCTGTGATTACCGACGACTACAAAGTGCCCAG**
CAAGAAATTCAAAGTCCTCGGAAACACCGATCGACACAGCATTAAGAAGAACCTCATCGGTGCCCTGCTCTTTGGCTCTGGAGAGACAGCAGAGGCGACACGCC
TCAAACGAACCGCGCGCCGACGATACACAAGGCGCAAGAACAGGATCTGCTATCTCCAAGAGATATTCTCTAATGAGATGGCGAAAGTGGACGACTCATTCTTT
CACCGACTGGAGGAGAGCTTTCTGGTGGAGGAGGACAAGAAACATGAGAGGCATCCGATCTTTGGCAATATTGTGGATGAGGTCGCATACCACGAGAAATACCC
CACAATCTATCATCTCCGTAAGAAACTGGCCGACTCTACCGACAAAGCCGACCTCCGACTGATCTATCTGGCACTGGCCCACATGATTAAGTTTAGGGGGCACT
TTCTGATTGAAGGTGACCTCAATCCTGACAATTCCGATGTCGATAAACTCTTTATCCAGCTGGTGCAGATATATAACCAACTGTTTGAAGAGAATCCTATTAAC
GCATCAAGGGTGGATGCTAAAGCTATCCTCTCGGCTAGGCTCTCGAAATCCCGACGACTCGAAAACCTCATTGCTCAACTCCCCGGCGAGAAACGAAATGGACT
GTTTGGGAATCTCATCGCCCTGTCCCTGGGGCTCACCCCCAACTTCAAATCAAATTTCGATCTCGCTGAGGACGCTAAACTCCAGCTCAGCAAAGATACATATG
ATGACGATCTGGACAATCTCCTGGCCCAAATCGGTGACCAATATGCTGATCTGTTTCTCGCAGCCAAGAACCTCAGCGACGCAATCCTGCTCTCAGACATTCTC
CGTGTTAATTCGGAAATTACTAAAGCACCGCTCAGCGCTAGCATGATTAAGCGCTACGACGAGCATCATCAAGACCTCACCCTGCTCAAAGCGCTGGTCCGTCA
ACAGCTGCCGGAGAAATATAAGGAAATCTTCTTTGATCAATCGAAGAATGGTTACGCGGGGTACATTGATGGTGGAGCGTCGCAAGAGGAGTTCTACAAATTCA
TTAAGCCAATTCTCGAAAAGATGGACGGAACGGAGGAACTCCTCGTCAAACTGAACCGCGAAGACTTGCTGCGGAAACAACGTACGTTTGACAACGGCTCAATT
CCCCATCAGATCCACTTGGGTGAGCTGCATGCGATCCTGCGCCGCCAGGAGGACTTCTATCCTTTCTTGAAAGACAATAGGGAGAAGATTGAGAAGATCCTCAC
TTTCAGGATTCCGTATTACGTGGGACCGTTGGCGCGTGGTAACAGCCGTTTCGCGTGGATGACCCGCAAATCCGAAGAAACGATTACTCCTTGGAACTTCGAGG
AAGTGGTGGACAAGGGTGCCTCAGCACAGTCGTTTATCGAACGGATGACGAACTTCGATAAGAACCTGCCCAACGAGAAAGTGCTGCCTAAACACTCGCTGCTG
TATGAGTACTTTACCGTGTATAATGAACTGACGAAAGTCAAGTACGTGACAGAAGGGATGCGCAAACCCGCGTTTCTCTCCGGAGAACAGAAGAAAGCAATCGT
GGACCTCTTGTTTAAGACGAACCGCAAAGTTACCGTGAAGCAGCTGAAAGAGGATTACTTTAAGAAGATAGAGTGTTTCGATTCAGTCGAGATTAGCGGGGTTG
AGGACAGGTTTAACGCGTCGCTGGGAGCATACCACGACCTGTTGAAGATAATTAAGGACAAGGATTTCCTGGACAACGAGGAGAATGAGGATATATTGGAGGAT
ATCGTGCTGACGTTGACATTGTTTGAGGACCGGGGCATGATCGAGGAGCGGTTGAAGACTTACGCCCACCTGTTTGACGATAAGGTTATGAAGCAGCTCAAACG
CCGGCGGTACACTGGTTGGGGCCGGCTGAGCCGCAAGCTGATTAACGGTATACGGGATAAACAGTCTGGAAAGACAATCCTCGACTTCCTGAAATCGGATGGGT
TCGCCAACAGGAATTTCATGCAGCTGATACACGATGATAGCCTGACTTTCAAGGAGGACATACAGAAAGCGCAGGTGTCTGGACAGGGGCACAGCCTCCACGAG
CAGATCGCAAATCTGGCCGGCTCTCCGGCAATCAAGAAAGGCATTCTCCAGACTGTGAAGATCGTCGATGAGCTGGTCAAAGTTATGGGGCACAAACCCGAGAA
CATAGTGATCGAGATGGCACGCGAGAATCAGACCACGCAGAAAGGACAGAAGAATAGCCGCGAGCGGATGAAACGGATCGAGGAGGGAATCAAGGAATTGGGCT
CGCAGATCCTGAAGGAGCACCCCGTCGAGAACACGCAGTTGCAGAACGAGAAGTTGTACCTCTACTACTTGCAGAATGGCCGGGACATGTACGTGGATCAGGAA
TTGGACATCAACAGGCTCTCTGACTACGATGTTGATCACATCGTGCCCCAGTCGTTCATCAAGGACGACAGCATCGATAACAAGGTGCTGACCCGCTCGGACAA
GAATCGCGGGAAGTCCGACAACGTGCCCTCTGAGGAAGTTGTTAAGAAGATGAAGAACTACTGGCGCCAGCTCCTGAACGCGAAGTTGATAACGCAGCGGAAGT
TCGACAACCTGACGAAGGCCGAGCGTGGCGGCCTGTCCGAGCTGGACAAGGCGGGGTTCATCAAGCGCCAGCTGGTGGAGACGCGCCAGATCACGAAGCACGTG
GCTCAGATCCTGGACTCGCGGATGAACACAAAGTACGACGAGAACGACAAGCTGATCCGCGAGGTGAAGGTCATCACATTGAAGTCCAAGCTCGTTTCCGACTT
CCGCAAGGACTTCCAGTTCTACAAGGTCAGGGAGATCAACAACTACCACCACGCTCACGACGCGTACCTGAACGCCGTGGTGGGCACGGCGCTCATCAAGAAGT
ACCCCAAGTTGGAATCTGAGTTCGTCTACGGGGACTACAAGGTGTACGACGTGCGGAAGATGATCGCCAAGAGCGAGCAGGAGATCGGCAAGGCCACGGCCAAG
TACTTCTTCTACTCGAACATCATGAACTTCTTCAAGACCGAGATCACACTCGCGAACGGGGAGATCAGGAAGCGCCCACTGATCGAGACGAACGGCGAGACCGG
CGAGATCGTGTGGGACAAGGGCCGCGACTTCGCCACCGTCCGTAAGGTGCTGAGCATGCCGCAGGTGAACATCGTCAAGAAGACGGAGGTGCAGACGGGCGGCT
TCAGCAAGGAGTCCATCTTGCCAAAGCGGAACAGCGACAAGCTCATCGCCCGTAAGAAGGACTGGGACCCAAAGAAGTACGGGGGCTTCGACAGCCCGACCGTG
GCGTACTCCGTGCTCGTCGTCGCCAAGGTGGAGAAGGGCAAGAGCAAGAAGCTGAAGTCGGTGAAGGAGTTGCTCGGCATAACGATCATGGAGCGTAGCAGCTT
CGAGAAGAACCCGATCGACTTCTTGGAAGCCAAGGGCTACAAAGAAGTTAAGAAGGACCTGATCATCAAGCTCCCAAAGTACTCCCTGTTCGAATTGGAGAACG
GGCGGAAGCGCATGCTGGCCAGCGCCGGCGAATTGCAGAAGGGCAACGAATTGGCCCTGCCATCGAAGTACGTCAACTTCCTGTACCTCGCGAGCCACTACGAG
AAGCTGAAGGGCTCCCCGGAGGACAACGAGCAGAAGCAGCTGTTCGTGGAGCAGCACAAGCACTACCTGGACGAGATCATCGAGCAAATATCGGAGTTCAGCAA
GCGCGTCATCCTGGCCGACGCCAACCTGGACAAGGTCCTGTCCGCGTACAACAAGCACAGGGACAAGCCAATCCGCGAGCAGGCCGAGAACATCATCCACCTGT
TCACCCTGACCAACCTCGGCGCCCCGGCCGCCTTCAAGTACTTCGACACGACCATCGACCGCAAGCGCTACACCTCCACCAAAGAAGTTCTGGACGCGACCCTG
ATCCACCAGAGCATCACGGGCCTGTACGAGACGCGCATCGACCTGTCCCAGCTCGGCGGCGACTccccagtgcggtcccccaagaagaaacggaaggtgtgatg
a

NLS

>LCas9-2

Globin 5’ UTR   Flag

taatacgactcactatagggagacaagcttgcttgttctttttgcagaagctcagaataaacgctcaactttggcagatctcgagccaccatggactacaagga

NLS

ccacgatggagactataaggaccacgacattgactacaaggatgacgacgataag atggcccccaagaagaaacgcaaggtgggcatccacggagtgcccgccg

Lamprey codon optimized Cas9-2

cg**ATGGACAAGAAATATTCTATTGGACTGGATATTGGGACCAACTCTGTGGGATGGGCGGTCATCACAGACGAGTACAAAGTCCCTTCCAAGAAATTCAAAGTG**
CTCGGGAACACTGATAGGCACTCCATTAAGAAGAACCTCATAGGAGCACTGCTCTTTGACAGCGGCGAGACCGCGGAGGCGACTCGGCTGAAGAGGACCGCCAG
GCGGCGCTACACACGACGAAAGAACCGAATTTGCTATCTGCAAGAAATCTTCTCTAACGAGATGGCCAAAGTGGATGATTCATTCTTTCATCGCCTGGAGGAGA
GCTTTCTGGTGGAGGAGGACAAGAAACACGAGCGCCACCCAATCTTTGGCAACATTGTCGATGAAGTCGCCTATCATGAGAAATATCCTACCATCTATCATCTG
CGTAAGAAACTGGTGGATAGCACAGACAAAGCTGACCTCCGGCTGATCTATCTGGCCCTGGCCCATATGATCAAGTTTCGCGGGCATTTCCTGATTGAGGGTGA
CCTCAACCCTGACAACTCCGATGTGGATAAACTGTTTATCCAACTCGTGCAGACATACAACCAGCTCTTTGAGGAGAACCCCATCAATGCTTCCGGCGTGGACG
CGAAAGCTATCCTCTCCGCTCGTCTGTCCAAATCGCGTAGGCTGGAGAACCTCATTGCGCAGCTGCCGGGTGAGAAGAAGAATGGTCTGTTTGGGAACCTGATC
GCTCTGTCCCTCGGTCTGACTCCTAACTTTAAGAGCAATTTCGATCTGGCCGAGGATGCAAAGCTGCAACTCTCCAAAGATACTTATGATGACGATCTGGATAA
TCTGCTCGCGCAGATCGGTGACCAGTATGCTGATCTGTTTCTCGCGGCTAAGAATCTCTCGGATGCCATTCTGCTCTCAGATATTCTGCGCGTCAACACAGAAA
TTACCAAAGCGCCGCTCAGCGCGTCCATGATCAAACGTTACGACGAGCATCATCAGGATCTGACTCTGCTGAAAGCTCTCGTGCGGCAACAACTCCCTGAGAAA
TATAAGGAAATATTCTTTGACCAGTCAAAGAACGGTTATGCAGGATATATAGATGGTGGAGCGAGCCAGGAGGAGTTCTACAAGTTTATCAAACCAATTCTCGA
AAAGATGGATGGCACCGAAGAACTGCTGGTGAAACTCAACAGGGAAGACCTGCTCCGTAAACAGCGCACCTTTGATAATGGCTCTATTCCGCATCAAATTCACC
TGGGAGAGTTGCACGCAATTCTCCGGCGCCAAGAGGATTTCTACCCATTCCTGAAAGACAATCGAGAGAAGATCGAGAAGATTCTGACGTTTCGTATTCCCTAC
TACGTCGGTCCACTGGCCCGCGGTAATTCTCGTTTCGCCTGGATGACGCGCAAATCCGAGGAAACTATAACCCCTTGGAATTTCGAGGAAGTGGTCGATAAAGG
CGCGTCTGCCCAGTCCTTCATCGAACGCATGACGAATTTCGACAAGAATCTCCCCAACGAGAAAGTGCTGCCGAAACACTCGCTCCTCTACGAGTACTTCACTG
TGTACAACGAGCTGACTAAAGTGAAATACGTCACGGAAGGAATGAGGAAACCAGCTTTCCTGTCAGGCGAACAGAAGAAAGCAATCGTGGATCTCTTGTTTAAG
ACGAACCGGAAAGTGACCGTCAAACAACTCAAAGAAGATTACTTTAAGAAGATAGAATGTTTCGATAGCGTCGAGATAAGCGGAGTCGAGGACAGGTTTAACGC
ATCACTCGGGACCTACCACGATCTCTTGAAGATAATCAAGGACAAAGACTTCCTGGATAACGAGGAGAACGAGGATATCCTGGAAGACATTGTGTTGACCCTCA
CACTGTTTGAGGACCGCGAGATGATCGAGGAACGACTGAAGACGTACGCCCACCTGTTTGACGACAAAGTCATGAAACAGCTGAAGCGACGACGGTACACGGGC
TGGGGCCGCCTCAGCCGTAAACTGATCAACGGCATCCGGGACAAGCAGAGCGGGAAGACGATATTGGACTTCTTGAAATCCGACGGCTTTGCGAATCGAAACTT
CATGCAGCTGATCCACGACGACAGCCTCACGTTCAAGGAGGACATCCAGAAAGCTCAGGTGAGCGGCCAGGGCGACTCGCTGCACGAGCACATTGCAAACCTGG
CTGGAAGCCCCGCCATCAAGAAGGGCATTCTGCAAACGGTTAAGGTCGTTGATGAACTGGTGAAGGTTATGGGGCGGCACAAGCCCGAGAACATCGTGATTGAG
ATGGCTCGCGAGAATCAAACCACGCAGAAGGGACAGAAGAACTCACGCGAGCGGATGAAGCGCATCGAGGAGGGGATCAAGGAGCTGGGATCACAGATTCTCAA
GGAGCACCCAGTCGAGAATACCCAGTTGCAGAATGAGAAGTTGTACTTGTACTACCTCCAGAACGGGCGCGACATGTACGTGGACCAGGAGCTGGACATTAACC
GTCTGTCGGACTACGACGTTGACCACATCGTGCCCCAGAGCTTCCTCAAGGACGACTCCATCGACAATAAGGTGTTGACGCGGTCTGACAAGAATCGCGGAAAG
AGCGACAATGTCCCGTCCGAAGAAGTTGTTAAGAAGATGAAGAATTACTGGCGGCAGCTCCTGAACGCCAAACTCATCACACAGAGGAAGTTCGACAACCTCAC
AAAGGCCGAGCGCGGCGGGCTCTCTGAATTGGACAAGGCGGGCTTCATCAAGCGCCAGTTGGTGGAGACGCGGCAGATCACAAAGCACGTGGCGCAGATCCTCG
ACTCCCGGATGAACACCAAGTACGACGAGAACGACAAGCTGATCCGGGAGGTGAAAGTTATAACGTTGAAGTCCAAGTTGGTCTCAGACTTCCGAAAGGACTTC
CAGTTCTACAAGGTGAGGGAGATCAACAACTACCACCACGCACACGACGCATACCTCAACGCGGTTGTTGGGACCGCCCTGATCAAGAAGTACCCCAAGCTGGA
GAGCGAGTTCGTGTACGGGGACTACAAGGTGTACGACGTGCGCAAGATGATCGCAAAGAGCGAGCAGGAGATCGGGAAGGCCACAGCCAAGTACTTCTTCTACT
CGAACATCATGAACTTCTTCAAGACGGAGATCACACTGGCGAATGGTGAAATACGGAAGAGGCCGCTCATCGAGACCAACGGCGAGACGGGGGAGATCGTGTGG
GACAAGGGGAGGGACTTCGCCACAGTGCGTAAAGTTCTCTCGATGCCCCAGGTGAACATCGTGAAGAAGACGGAGGTGCAGACCGGCGGCTTCTCTAAGGAGTC
CATCCTGCCGAAGCGGAACAGCGACAAGTTGATCGCACGCAAGAAGGACTGGGACCCGAAGAAGTACGGCGGCTTCGACTCACCGACCGTCGCCTACAGCGTGC
TGGTCGTGGCCAAGGTGGAGAAGGGGAAGTCGAAGAAGCTCAAGTCGGTCAAGGAATTGCTCGGCATCACGATCATGGAGCGCAGCTCGTTCGAGAAGAACCCG
ATCGACTTCTTGGAAGCGAAGGGCTACAAAGAAGTTAAGAAGGACCTGATCATCAAGCTGCCGAAGTACTCGCTGTTCGAGCTGGAGAACGGGAGGAAGCGCAT
GCTGGCCTCGGCAGGCGAGTTGCAGAAAGGCAACGAGCTGGCGCTCCCCTCGAAGTACGTGAACTTCTTGTACTTGGCCTCGCACTACGAGAAGCTCAAGGGCA
GCCCAGAGGACAACGAGCAGAAGCAGCTCTTCGTGGAGCAGCACAAGCACTACTTGGACGAGATCATCGAGCAAATATCGGAGTTCAGCAAGCGCGTGATCTTG
GCGGACGCCAACCTGGACAAGGTCCTGTCGGCCTACAACAAGCACCGCGACAAGCCCATCCGAGAGCAGGCCGAGAACATCATCCACCTCTTCACCCTCACGAA
CCTGGGCGCACCAGCGGCCTTCAAGTACTTCGACACGACCATCGACCGCAAGAGGTACACGAGCACCAAAGAAGTCTTGGACGCCACGCTGATCCACCAGAGCA
TCACGGGCTTGTACGAGACGCGCATCGACCTGTCCCAGCTGGGCGGCGACaagaggcccgcggccaccaagaaggcgggccaggccaagaagaagaaatga

NLS

Figure S1. The DNA sequences of LCas9-1 and LCas9-2.

Figure S2.

wee1

CCTCACGGTGGCAAAAGTACAACGTGGGCGAATTTTAAACTTTTTTTTACTTTAAAAAGTTTCAACAACAAAAACATTGGGATAAGAAGAC
GACGATAAGAACGAGAAAACGTCGCCCGATTTTTAAAACAACAATGAGCCGATTCTCGACATTTACGAGAGTTTCCGCCAAGCTGGATTACCT
CAGCGACGACGAAGAAGGCCCAGGTACACCTCCCGACCGAGAGGCCTCAAGCCTCGGCCTCGCCTTGACACCTCCGCCGGGCGCCTCGCAC
CGAAGCCGGCGGGTCGTCCAGGCCACGGCGGGCTTCTTGGGCGGGAGTCGCGAGGCGCTGTCGTGTTGGGACG**CGTCGATGGAGTCTCCG
TCGCCCGTGAAG**

soxe2

TGTCGGATATCAATCACGAGATGTCGCGCACTCCGAGCCCGCACTGCTCCTCGGACGCCGGGTCTGGACTCGGATCCCCTGGCCGCAGCGA
TCCCGAGCCGAGCTCCGTCGAGCACGGAGGAGGCGGCGGCGGCGGAGGAGGAGGAGGCTCGGGTGGCTGCAGCTCATCGGGCTCGGA
GCCGTCGAGGGGAGGAGGCACCGACGACCCCTTCTCGGAGAGCATCCAGGCGGCCGTGAGTCAAGTGCTGGACGGCTACGACTGGTCGCTG
TTGCCCGTGCCCGTGCGTGGAGCCGGTGGGCCGGGGGGATGCAAGCCGGGCGAGAAGCCCCACGTGAAGAGACCCATGAACGCGTTCATGG
TGTGGGCGCAAGCGGCTCGCAGG**AAGCTGTCCGACCAATACCC**

kctd10

ACATCCAGACTCGTGTTCCTTCTGCGCACCGCCAAGATTGCCCG-TCAGCCACGTGCCGCTTCACGCAACGAGCCGCCTTTGTCGGGTACCC
CCGTGTTCACGCGAGCGCACCGCGCCTCTCTCCCACTCTCACCGCGACGCCGGCGAGCAGGCTGGATCCTGATCGACCGCTGCGGAAAGCAC
TTTGGCACGATCCTCAACTTCCTGCGCGACGGCGCCGTGCCCCTGCCGGAGTCTCGCACCGAGATCGAGGAGCTGCTCACAGAGGCCAAGTA
CTACCTGGTGCAAGGCCTGGGACAGCAGTGCCACGCCGCCCT**GCAGGTATCATCGCGTATTTC**

wnt7b

TTATCGCCGTTTCCACTCCACCGACCAGGTGCTGGAGGAGAAAATGAAGCTGGAGTGTAAGTGCCACGGCGTGTCGGGCTCGTGCACCAC
CAAGACGTGCTGGACCACGCTGCCCAAGTTCCGCGAGGTGGGCTACGCGCTCAAGGACAAGTACGAGCACACGGTGCACGTGGAGCCGGTG
AGCGGCAGCCGTTACCTGCGGCCGATGTTCCTCAAGGTGAAGAAGTCGCGCTCGTACCGCAAGCCGCTCGACACGGAGCTCGTGTTCATCGA
GCGCTCGCCCAACTACTGCGAGGAGGACCCGGCTACGGGCAGCGCGGGGACACGCGGTCGCCTGTGTAACCGCACGTCGCCGCACGCTGAC
GGCTGCGACCTTCTGTGCTGCGGCCGC**GGCTACAACACGCACCAGTA**

golden

CTCTCGCTCGTGTCCGTCTACGCTGCCTACGTGGTGGCTCTGCACTTCGACGCGCGCCTCCGCCGCGCCTTCACACGCGGCTGCTTGTGCCT
CCGCACGGCCGGGCAGCACCGCTGCCATCAGCCGGGTCACGACCCCGAGACGCAGCCGCTCGTCGGCGTCGACGCGGCCGGCGAGGAACA
CAGT-GGCGGCACCTCGCGTCGCTCCTCGACGGGGGTCAACGACTTCCTGCACGGGCGCGCCAGGTCGGACAGCGGCGTCATCCTCAAGGAC
GGCTCGGACTACT**CGCAGTTGACGCTCAGCCTGCACGG**

Figure S2. The sequences of the target regions of five genes of lamprey. The target sites of each gene are labeled with box, and the primer pairs for amplifying each target region are underlined and in bold.

Figure S3.

```
SLC24A5_human       1   MQTK-GGQTWARR--------ALLLGILWATAHLPLSGTSLP----QRLPRATGN 42
SLC24A5_mouse       1   MRTK-WGPTWTRR--------VLLLGIFWVSAYLPVRGVSLP----PRLPRATGN 42
Slc24a5_zebrafish   1   MRTDVFLQRRKRRDV-LLSIIALLLLIFAIVHLVFCAGLSFQGSSSARVRRDLEN 54
Slc24a5_lamprey     1   MDPS-LGSSAARCILGASSTVLLLLRLDYAVGLEAGAGAHHSNHMRVRRDEETGN 54

SLC24A5_human       43  STQCVISPSSEFFEGFFTRQERRDGGIIIYFLIIVYMFMAISIVCDEYFLPSLEI 97
SLC24A5_mouse       43  STQCAVSPASEFFEGFFTKQESTDGGIVIYFLIILYMCMAISIVCDKYFLPSLEI 97
Slc24a5_zebrafish   55  ASECVQPQSSEFFEGFFTVQERKDGGILIYFMIIFYMLLSVSIVCDEYFLPSLEV 109
Slc24a5_lamprey     55  DTGCVGPASDEFFPGFFTPEEQREGGVLIYILIALYLLLAIAIVCDYYFLPSLEL 109

SLC24A5_human       98  ISESLGLSQDVAGTTFMAAGSSAPELVTAFLGVFITKGDIGISTILGSAIYNLLG 152
SLC24A5_mouse       98  ISDSLGLSQDVAGATFMAAGSSAPELVTAFLGVFITKGDIGISTILGSAIYNLLG 152
Slc24a5_zebrafish   110 ISERLGLSQDVAGATFMAAGSSAPELVTAFLGVFVTKGDIGVSTIMGSAVYNLLC 164
Slc24a5_lamprey     110 ISDRLGLTMDVAGATFMAAGSSAPEMVTAFLGVFVTKGDIGVSTIVGSAVYNLLG 164

SLC24A5_human       153 ICAACGLLSNTVSTLSCWPLFRDCAAYTISAAAVLGIIYDNQVYWYEGALLLLIY 207
SLC24A5_mouse       153 ICAACGLLSNMVSTLSCWPLFRDCAVYAVSVGAVFGIIFDNRIYWYEGAGLLLIY 207
Slc24a5_zebrafish   165 ICAACGLLSSAVGRLSCWPLFRDCVAYSISVAAVIAIISDNRVYWYDGACLLLVY 219
Slc24a5_lamprey     165 ICAICCLFSPSASPLSHWPLLRDSLAYAVSVAVLIGCIHDGKVHWYEALSLVSVY 219

SLC24A5_human       208 GLYVLVLCFDIKINQYIIKKCSPCCACLAKAME--------RSEQQPLMGWE--- 251
SLC24A5_mouse       208 GLYVLLLCFDTTISRHVMKTCSPCCPCLARAMEE-------RIEQQTLLGWE--- 252
Slc24a5_zebrafish   220 GVYVAVLCFDLKISEYVMQRFSPCCWCLKPRDRD-------SGEQQPLVGWS--- 264
Slc24a5_lamprey     220 AAYVVVLRFDARLHR----AFTRGCLCLRPAGQHRRHQPGHDPETQPLVGVDAAG 270

SLC24A5_human       252 -DEGQPFIRR-----------QSRTDSGIFYED-SGYSQLSISLHGLSQVS-EDP 292
SLC24A5_mouse       253 -DESQLFIRR-----------QSRTDSGIFQED-SGYSQLSLSLHGLSQVS-EDP 293
Slc24a5_zebrafish   265 -DDSSLRVQR-----------RSRNDSGIFQDD-SGYSHLSLSLHGLNEIS-DEH 305
Slc24a5_lamprey     271 EEHSGGTSRRSSTGVNDFLHGRTRSDSGVILRDGSDYSQLTLSLHGLTSLEDEEP 325

SLC24A5_human       293 PSVFNMPEADLKRIFWVLSLPIITLLFLTTPDCRKKFWKNYFVITFFMSAIWISA 347
SLC24A5_mouse       294 PSVFSMPEADLRRIFWVLSLPIITLLALTTPDCRRKFWKNYFVITFFMSALWISA 348
Slc24a5_zebrafish   306 KSVFSMPDHDLKRILWVLSLPVSTLLFVSVPDCRRPFWKNFYMLTFLMSAVWISA 360
Slc24a5_lamprey     326 MGLLHVPQGDGRRVLWVLSLPVLLLLTLTVPDCRARSMRRFFPVTFFMAAVWISA 380

SLC24A5_human       348 FTYILVWMVTITGETLEIPDTVMGLTLLAAGTSIPDTIASVLVARKGKGDMAMSN 402
SLC24A5_mouse       349 FTYILVWMVTVTGETLGIPDTVMGLTLLAAGTSIPDTVTSVLVARKGKGDMAISN 403
Slc24a5_zebrafish   361 FTYVLVWMVTIVGETLGIPDTVMGMTLLAAGTSIPDTVASVMVAREGKSDMAMSN 415
Slc24a5_lamprey     381 LTYLLVWMVTVTGETLGIPDTVMGLTLLAIGTSLPDTITSLLVAREGKADMAMSN 435

SLC24A5_human       403 IVGSNVFDMLCLGIPWFIKTAFINGSAPAEVNSRGLTYITISLNISIIFLFLAVH 457
SLC24A5_mouse       404 IVGSNVFDMLCLGLPWFIKTAFTNASAPIEVNSKGLTYITISLNISILFLFLAVH 458
Slc24a5_zebrafish   416 IVGSNVFDMLCLGLPWFIQTVFVDVGSPVDVNSSGLVFMSCTLLLSIIFLFLAVH 470
Slc24a5_lamprey     436 IVGSNVFDMLCLGLPWLLLTAFVSPSGPAHVSSAGLVYSASSLLISLALLLACVH 490

SLC24A5_human       458 FNGWKLDRKLGIVCLLSYLGLATLSVLYELGIIGNN-KIRGCGG 500
SLC24A5_mouse       459 FNGWKLDRKLGVVCLVLYLGLATLSVLYEIGIIGNN-RIRGCGV 501
Slc24a5_zebrafish   471 INGWKLDWKLGLVCLACYILFATLSILYELGIIGNN-PIRSCSD 513
Slc24a5_lamprey     491 AARWRLSRPLGAACLAAYLVFVAVAVLHEVGVLGGDPATRVCGK 534
```

Figure S3. The alignment of the sequences of Slc24a5 (encoded by the gene of *golden*) among human, mouse, zebrafish and lamprey. The target site of Cas9-gRNA is labeled with the red box.

Figure S4.

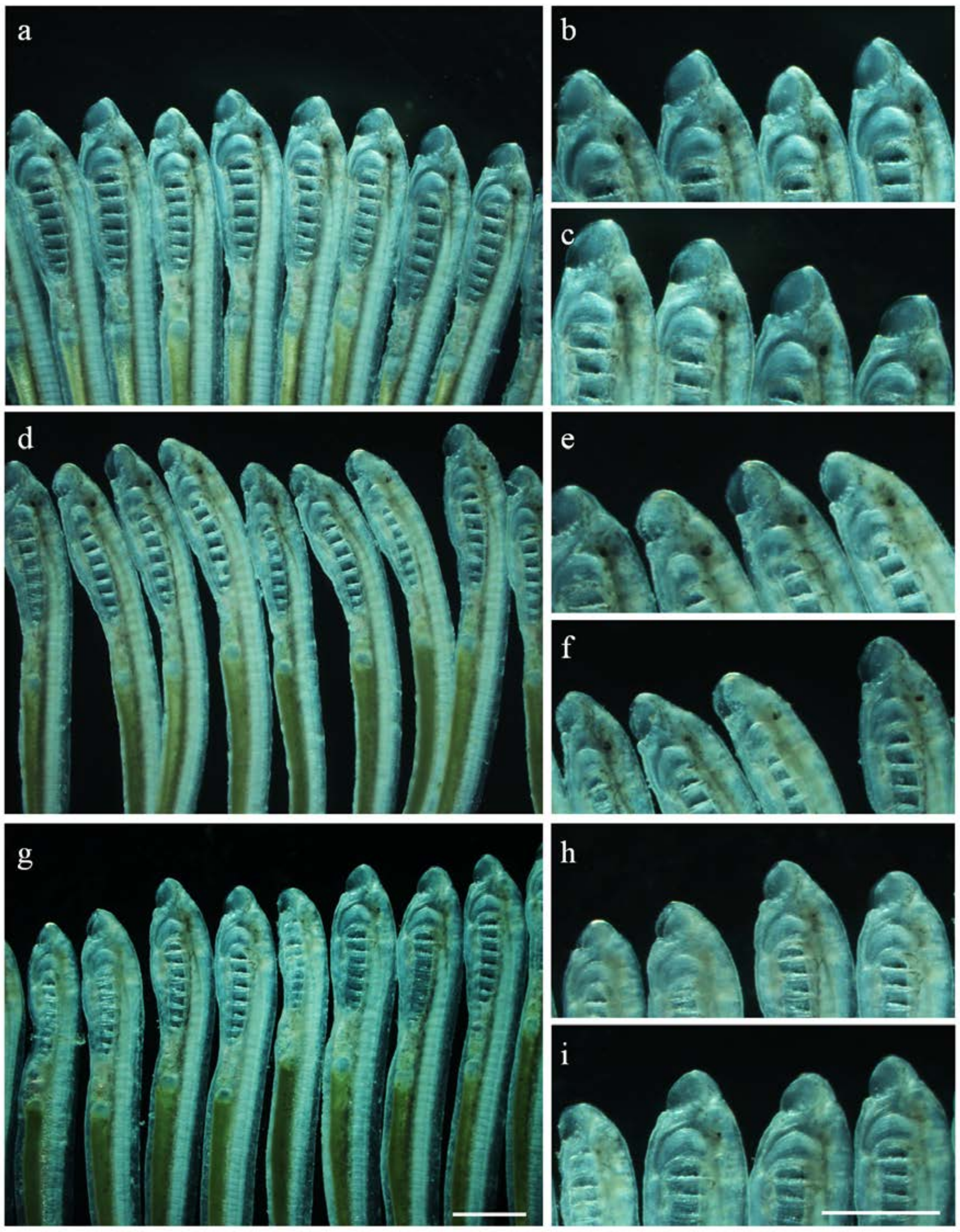

Figure S4. Injection of Cas9 and gRNA against *golden* resulting in mosaic and null pigmentation in the retinas caused by biallelic disruption of this gene. Lateral views of control lamprey larvae (**a-c**) and *gol* gRNA injected larvae (**d-i**) at 19 dpf. Some larvae injected with *Cas9* mRNA and *gol* gRNA showed mosaic hypopigmentation (**d-f**), some larvae were almost unpigmented in their retinas (**g-i**). (Scale bars: 1mm)

Figure S5.

A

N2

```
Reference    GGAACACAGTGGCGGCACCTCGCGTCGCTCCTCGACGGGGGTCAACGACTTCCTGCACGGGCGCGCCAGGTCGGACAGCGGCGTCATC
Seq1:        GGAACACAGTGGCGGCACCTCGCGTCGCTCCTCGACGGGGGTCAACGAC---------GGGCGCGCCAGGTCGGACAGCGGCGTCATC（-9bp）
Seq2:        GGAACACAGTGGCGGCACCTCGCGTCGCTCCTCGACGGGGGTCAACGAC---------GGGCGCGCCAGGTCGGACAGCGGCGTCATC(-9bp)
Seq3:        GGAACACAGTGGCGGCACCTCGCGTCGCTCCTCGACGGGGGTCAACGAC---------GGGCGCGCCAGGTCGGACAGCGGCGTCATC（-9bp）
Seq4:        GGAACACAGTGGCGGCACCTCGGGTCGCTCCTCGACGG------------------------GCGCCAGGTCGGACAGCGGCGTCATC(-24bp)
Seq5:        --------------------------------------------------------------------------------------TC(-111bp)
Seq6:        GGAACACAGTGGCGGCACCTCGCGTCGCTCCTCGACGGGGGTCAACGACTTCCTGT-CGGGCGCGCCAGGTCGGACAGCGGCGTCATC(-2, +1bp)
Seq7:        GGAACACAGTGGCGGCACCTCGCGTCGCTCCTCGACGGGGGTCAACGACTTCCTGT-CGGGCGCGCCAGGTCGGACAGCGGCGTCATC(-2, +1bp)
Seq8:        GGAACACAGTGGCGGCACCTCGCGTCGCTCCTCGACGGGGGTCAACGACTTCC-----GGGCGCGCCAGGTCGGACAGCGGCGTCATC(-5bp)
Seq9:        GGAACACAGTGGCGGCACCTCGCGTCGCTCCTCGACGGGGGTCAACGACTTCC-----GGGCGCGCCAGGTCGGACAGCGGCGTCATC(-5bp)
Seq10:       GGAACACAGTGGCGGCACCTCGCGTCGCTCCTCGACGGGGGTCAACGACTTCC-----GGGCGCGCCAGGTCGGACAGCGGCGTCATC(-5bp)
Seq11:       GGAACACAGTGGCGGCACCTCGCGTCGCTCCTCGACGGGGGTCAACGACTTCC-----GGGCGCGCCAGGTCGGACAGCGGCGTCATC(-5bp)
Seq12:       GGAACACAGTGGCGGCACCTCGCGTCGCTCCTCGACGGGGGTCAACGACTTCC-----GGGCGCGCCAGGTCGGACAGCGGCGTCATC(-5bp)
Seq13:       GGAACACAGTGGCGGCACCTCGCGTCGCTCCTCGACGGGGGTCAACGACTTCC-----GGGCGCGCCAGGTCGGACAGCGGCGTCATC(-5bp)
Seq14:       GGAACACAGTGGCGGCACCTCGCGTCGCTCCTCGACGGGGGTCAACGACTTCC-----GGGCGCGCCAGGTCGGACAGCGGCGTCATC(-5bp)
Seq15:       GGAACACAGTGGCGGCACCTCGCGTCGCTCCTCGACGGGGGTCAACGACTTCC-----GGGCGCGCCAGGTCGGACAGCGGCGTCATC(-5bp)
Seq16:       GGAACACAGTGGCGGCACCTCGCGCCGCTCCTCGACGGGGGTCAACGACTTCCTGC--------GCCAGGTCGGACAGCGGCGTCATC(-8bp)
Seq17:       GGAACACAGTGGCGGCACCTCGCGTCGCTCCTCGACGG--------------------------------TCGGACAGCGGCGTCATC(-32bp)
Seq18:       GGAACACAGTGGCGGCACCTCGCGTCGCTCCTCGACGG--------------------------------TCGGACAGCGGCGCCATC(-32bp)
```

N5

```
Reference    GGAACACAGTGGCGGCACCTCGCGTCGCTCCTCGACGGGGGTCAACGACTTCCTGCACGGGCGCGCCAGGTCGGACAGCGGCGTCATC
Seq1:        GGAACACAGTGGCGGCACCTCGCGTCGCTCCTCGACGGGGGTCAACGAC---------GGGCGCGCCAGGTCGGACAGCGGCGTCATC(-9bp)
Seq2:        GGAACACAGTGGCGGCACCTCGCGTCGCTCCTCGACGGGGGTCAACGAC---------GGGCGCGCCAGGTCGGACAGCGGCGTCATC(-9bp)
Seq3:        GGAACACAGTGGCGGCACCTCGCGTCGCTCCTCGACGGGGGTCAACG------------------CCAGGTCGGACAGCGGCGTCATC(-18bp)
Seq4:        GGAACACAG---------------------------------------------------GCGCGCCAGGTCGGACAGCGGCGTCATC(-51bp)
Seq5:        GGAACACAGCGGCGGCACCTCGCGTCGCTCCTCGACGGGGGTCAACGACTTCCTG----GGCGCGCCAGGTCGGACAGCGGCGTCATC(-4bp)
Seq6:        GGAACACAGTGGCGGCACCTCGCGTCGCTCCTCGACGGGGGTCAACGACTTCCTG----GGCGCGCCAGGTCGGACAGCGGCGTCATC(-4bp)
Seq7:        GGAACACAGTGGCGGCACCTCGCGTCGCTCCTCGACGGGGGTCAACGACTTCC----CGGGCGCGCCAGGTCGGACAGCGGCGTCATC(-4bp)
Seq8:        GGAACACAGTGGCGGCACCTCGCGTCGCTCCTCGACGGGGGTCAACGACTTCCTGC-----GGCGTCAGGTCGGACAGCGGCGTCATC(-5bp)
Seq9:        GGAACACAGTGGCGGCACCTCGCGTCGCTCCTCGACGGGGGTCAACGAC-------------GCGCCAGGTCGGACAGCGGCGTCATC(-13bp)
Seq10:       GGAACACAGTGGCGGCACCTCGCGTCGCTCCTCGACGGGGGTCAACGAC-------------GCGCCAGGTCGGACAGCGGCGTCATC(-13bp)
Seq11:       GGAACACAGTGGCGGCACCTCGCGTCGCTCCTCGACGGGGGTCAACGAC-------------GCGCCAGGTCGGACAGCGGCGTCATC(-13bp)
Seq12:       GGAACACAGTGGCGGCACCTCGCGTCGCTCCTCGACGGGGGTCAACGAC-------------GCGCCAGGTCGGACAGCGGCGTCATC(-13bp)
Seq13:       GGAACACAGTGGCGGCACCTCGCGTCGCTCCTCGACGGGGG--------------------CGCGCCAGGTCGGACAGCGGCGTCATC(-20bp)
Seq14:       GGAACACAGTGGCGGCACCTCG-------------------------------------GGCGCGCCAGGTCGGACAGCGGCGTCATC(-37bp)
Seq15:       GGAACACAGTGGCGGCACCTCGCGTC-----------------------------------------------------------ATC（-59bp）
Seq16:       GGAACACAGTGGCGGCACCTCGCGTCGCTCCTCGACGGGCGTCACCGACTTCCGTCCTTGACCCCCGGGCGCGCCAGGTCGGACAGCG(-4,+12bp)
Seq17:       GGAACACAGTGGCGGCACCTCGCGTCGCTCCTCGACGGGGGTCAACGACTTCCTGCACGGGCGCGCCAGGTCGGACAGCGGCGTCATC(wt)
```

B

M2

```
Reference    GGAACACAGTGGCGGCACCTCGCGTCGCTCCTCGACGGGGGTCAACGACTTCCTGCACGGGCGCGCCAGGTCGGACAGCGGCGTCATC
Seq1:        GGAACACAGTGGCGGCACCTCGCGTCGCTCCTCGACGGGGGTCAACGAC---------GGGCGCGCCAGGTCGGACAGCGGCGTCATC(-9bp)
Seq2:        GGAACACAGTGGCGGCACCTCGCGTCGCTCCTCGACGGGGATCAACG---------ACGGGCGCGCCAGGTCGGACAGCGGCGTCATC(-9bp)
Seq3:        GGAACACAGTGGCGGCACCTCGCGTCGCTCCTCGACGGGGGTCAACGAC---------------GCCAGGTCGGACAGCGGCGTCATC(-15bp)
Seq4:        GGAACACAGTGGCGGCACCTCGCGTCGCTCCTCGACGGGGGTCAACGAC---------------GCCAGGTCGGACAGCGGCGTCATC(-15bp)
Seq5:        GGAACACAGTGGCGGCACCTCGCGTCGCTCCTCGACGGGGGTCAACGAC---------------GCCAGGTCGGACAGCGGCGTCATC(-15bp)
Seq6:        GGAACACAGTGGCGGCACCTCGCGTCGCTCCTCGACGGGGGTCAACGAC---------------GCCAGGTCGGACAGCGGCGTCATC(-15bp)
Seq7:        GGAACACAGTGGCGGCACCTCGCGTCGCTCCTCGACGGGGGTCAACGAC---------------GCCAGGTCGGACAGCGGCGTCATC(-15bp)
Seq8:        GGAACACAGTGGCGGCACCTCGCGTCGCTCCTCGACGGGGGTCAACGACGGCC---------------GGTCGGACAGCGGCGTCATC(-19,+4bp)
Seq9:        GGAACACAGTGGCGGCACCTCGCGTCGCTCCTCGACGGGGGTCA------------------------GGTCGGACAGCGGCGTCATC(-24bp)
Seq10:       GGAACACAGTGGCGGCACCTCGCGTCGCTCCTCGACGGGGGTCAACGACTTCCT---------------------------------- （-36bp）
Seq11:       GGAACACAGTGGCGGCACCTCGCGTCGCTCCTCGACGGGGGTCAACGACTTCC-----GGGCGCGCCAGGTCGGACAGCGGCGTCATC(-5bp)
Seq12:       GGAACACAGTGGCGGCACCTCGCGTCGCTCCTCGACGGGGGTCAACGAC----------GGCGCGCCAGGTCGGACAGCGGCGTCATC(-10bp)
Seq13:       GGAACACAGTGGCGGCACCTCGCGTCGCTCCTCGACGGGGGTCAACGAC----------GGCGCGCCAGGTCGGACAGCGGCGTCATC(-10bp)
Seq14:       GGAACACAGTGGCGGCACCTCGCGTCGCTCCTCGACGGGGGTCA-------------CGGGCGCGCCAGGTCGGACAGCGGCGTCATC(-13bp)
Seq15:       GGAACACAGTGGCGGCACCTCGCGTCGCTCCTCGACGGG----------------------CGCGCCAGGTCGGACAGCGGCGTCATC(-22bp)
```

```
Seq16:    GGAACACAGTGGCGGCACCTCGCGTCGCTCCTCGACGGGGGTC-----------------------------GGACAGCGGCGTCATC(-29bp)
Seq17:    GGAACACAGTGGCGGCACCTCGCGTCGCTCCTCGACGGGGGTCAACG-----------------------------------------(-56bp)
Seq18:    GGAACACAGTGGCGGCACCTCGCGTCGCTCCTCGACGGGGGTCAACG-----------------------------------------(-56bp)
```

M5

```
Reference GGAACACAGTGGCGGCACCTCGCGTCGCTCCTCGACGG[GGGTCAACGACTTCCTGCAC]GGGCGCGCCAGGTCGGACAGCGGCGTCATC
Seq1:     GGAACACAGTGGCGGCACCTCGCGTCGCTCCTCGACGGGGGTCAACGACTTCC---ACGGGCGCGCCAGGTCGGACAGCGGCGTCATC(-3bp)
Seq2:     GGAACACAGTGGCGGCACCTCGCGTCGCTCCTCGACGGGGGTCAACGACTTCC---ACGGGCGCGCCAGGTCGGACAGCGGCGTCATC(-3bp)
Seq3:     GGAACACAGTGGCGGCACCTCGCGTCGCTCCTCGACGGGGGTCAACGACTTCC---ACGGGCGCGCCAGGTCGGACAGCGGCGTCATC(-3bp)
Seq4:     GGAACACAGTGGCGGCACCTCGCGTCGCTCCTCGACGGGGTTCAACGACTTCCTGCA---------CAGGTCGGACAGCGGCGTCATC(-9bp)
Seq5:     GGAACACAGTGGCGGCACCTCGCGTCGCTCCTCGACGGGGGTCAACG------------GGCGCGCCAGGTCGGACAGCGGCGTCATC(-12bp)
Seq6:     GGAACACAGTGGCGGCACCTCGCGTCGCTCCTCGACGGGGGTCAACGACT------------------GGTCGGACAGCGGCGTCATC(-18bp)
Seq7:     GGAACACAGTGGCGGCACCTCGCGTCGCTCCTCGACGGGGGTCAACG------------------CCAGGTCGGACAGCGGCGTCATC(-18bp)
Seq8:     GGAACACAGTGGCGGCACCTCGCGTCGCTCCTCGACGGGGGTCAACGACTT----CACGGGCGCGCCAGGTCGGACAGCGGCGTCATC(-4bp)
Seq9:     GGAACACAGTGGCGGCACCTCGCGTCGCTCCTCGACGGGGGTCAACGACTTC----ACGGGCGCGCCAGGTCGGACAGCGGCGTCATC(-4bp)
Seq10:    GGAACACAGTGGCGGCACCTCGCGTCGCTCCTCGACGGGGGTCAACGACTTCCTG----GGCGCGCCAGGTCGGACAGCGGCGTCATC(-4bp)
Seq11:    GGAACACAGTGGCGGCACCTCGCGTCGCTCCTCGACGGGGGTCAACGACTTCCTG----GGCGCGCCAGGTCGGACAGCGGCGTCATC(-4bp)
Seq12:    GGAACACAGTGGCGGCACCTCGCGTCGCTCCTCGACGGGGGTCAACGACTTCCTGC--------GCCAGGTCGGACAGCGGCGTCATC（-8bp）
Seq13:    GGAACACAGTGGCGGCACCTCGCGTCGCTCCTCG-------------------------------CCAGGTCGGACAGCGGCGTCATC(-31bp)
Seq14:    GGAACACAGTGGCGGCACCTCGCGTCGCTCCTCG-------------------------------CCAGGTCGGACAGCGGCGTCATC(-31bp)
Seq15:    GGAACACAGTGGCGGCACCTCGCGTCGCTCCTCG-------------------------------CCAGGTCGGACAGCGGCGTCATC(-31bp)
Seq16:    GGAACACAGTGGCGGCACCTCGCGTCGCTCCTCG-------------------------------CCAGGTCGGACAGCGGCGTCATC(-31bp)
```

C

W5

```
Reference GGAACACAGTGGCGGCACCTCGCGTCGCTCCTCGACGG[GGGTCAACGACTTCCTGCAC]GGGCGCGCCAGGTCGGACAGCGGCGTCATC
Seq1:     GGAACACAGTGGCGGCACCTCGCGTCGCTCCTCGACGGGGGTCAACGACTTC------GGGCGCGCCAGGTCGGACAGCGGCGTCATC(-6bp)
Seq2:     GGAACACAGTGGCGGCACCTCGCGTCGCTCCTCGACGGGGGTCAACGAC---------GGGCGCGCCAGGTCGGACAGCGGCGTCATC(-9bp)
Seq3:     GGAACACAGTGGCGGCACCTCGCGTCGCTCCTCGACGAGGGTCAACGA------------------CAGGTCGGACAGCGGCGTCATC(-18bp)
Seq4:     GGAACACAGTGGCGGCACCTCGCGTCGCTCCTCGACGGGGGTCAAC---------------------AGGTCGGACAGCGGCGTCATC(-21bp)
Seq5:     GGAACACAGTGGCGGCACCTCGCGTCGCTCCTCGACGGGGGTCAACGAC---------------------------AGCGGCGTCATC(-27bp)
Seq6:     GGAACACAGTGGCGGCACCTCGCGTCGC------------------------------------GCCAGGTCGGACAGCGGCGTCATC(-36bp)
Seq7:     GGAACACAGTGGCGGCACCTCGCGTCGC------------------------------------GCCAGGTCGGACAGCGGCGTCATC(-36bp)
Seq8:     ---------------------------------------------------------CGGGCGCGCCAGGTCGGACAGCGGCGTCATC(-87bp)
Seq9:     ---------------------------------------------------------CGGGCGCGCCAGGTCGGACAGCGGCGTCATC(-87bp)
Seq10:    ---------------------------------------------------------CGGGCGCGCCAGGTCGGACAGCGGCGTCATC(-87bp)
Seq11:    GGAACACAGTGGCGGCACCTCGCGTCGCTCCTCGACGGGGGTCAACGACTTCCTCGTGGGGCGCGCCAGGTCGGACAGCGGCGTCATC(-4,+4bp)
Seq12:    GGAACACAGTGGCGGCACCTCGCGTCGCTCCTCGACGGGGGTCAACGACTTCCTCGTGGGGCGCGCCAGGTCGGACAGCGGCGTCATC(-4,+4bp)
Seq13:    GGAACACAGTGGCGGCACCTCGCGTCGCTCCTCGACGGGGGTCAACGACTTCCTCGTGGGGCGCGCCAGGTCGGACAGCGGCGTCATC(-4,+4bp)
Seq14:    GGAACACAGTGGCGGCACCTCGCGTCGCTCCTCGACGGGGGTCAACGACTTCC--------------AGGTCGGACAGCGGCGTCATC(-14bp)
Seq15:    GGAACACAGTGGCGGCACCTCGCGTCGCTCCTCGACGGG----------------------CGCGCCAGGTCGGACAGCGGCGTCATC(-22bp)
Seq16:    GGAACACAGTGGCGGCACCTCGCGTCGCTCCTCGACGGGGGTCAACG--------------------------GACAGCGGCGTCATC(-26bp)
Seq17:    GGAACACAGTGGCGGCACCTCGCGTCGCTCCTCGACGGG-------------------------------TCGGACAGCGGCGTCATC(-31bp)
```

Figure S5. The sequences of the target region of *gol* in the lamprey larvae injected with Cas9 and *gol* gRNA. **A**, The sequences of the target region of *gol* in the injected lamprey larvae with no pigment in the retinas; **B**, The sequences of the target region of *gol* in the injected lamprey larvae with mosaic pigment in the retinas; **C**, The sequences of the target region of *gol* in the injected lamprey larvae with normal pigment in the retinas. The target sites are labeled with box, the PAM sequences are highlighted with yellow and insertions are highlighted with red.

Figure S6.

```
KCTD10_human      1    MEEMS-GES---VVSSAVP-----AAAT------RTTSFKGTSPSSKYVKLNVGGA 41
KCTD10_mouse      1    MEEMS-GDS---VVSSAVP-----AAAT------RTTSFKGASPSSKYVKLNVGGA 41
Kctd10_zebrafish  1    MEEMS-GES---VVSSAVP-----AATT------RTTSFKGSSPSSKYVKLNVGGA 41
kctd10_lamprey    1    MSAQGPGHAPAGPLSVSVPPLPPMAAGTAQPGTGRAVSFKGVSPSSKYVKLNVGGA 56

KCTD10_human      42   LYYTTMQTLTKQDTMLKAMFSGRMEVLTDSEGWILIDRCGKHFGTILNYLRDGAVP 97
KCTD10_mouse      42   LYYTTMQTLTKQDTMLKAMFSGRMEVLTDSEGWILIDRCGKHFGTILNYLRDGGVP 97
Kctd10_zebrafish  42   LYYTTMQTLTKQDTMLKAMFSGRMEVLTDSEGWILIDRCGKHFGTILNYLRDGVVP 97
kctd10_lamprey    57   LFYTTMQTLTKQDTMLKAMFSGRMEVLTDSEGWILIDRCGKHFGMILNFLRDGAVP 112

KCTD10_human      98   LPESRREIEELLAEAKYYLVQGLVEECQAALQNK-DTYEPFCKVPVITSSKEEQKL 152
KCTD10_mouse      98   LPESRREIEELLAEAKYYLVQGLLEECQAALQNK-DTYEPFCKVPVITSSKEEQRL 152
Kctd10_zebrafish  98   LPESRRETEELLAEAKYYLVQGLVDECQAALQNK-DAYEPFCKVPLVTSSKEEQRL 152
kctd10_lamprey    113  LPESRTEIEELLTEAKYYLVQGLGQQCHAALQQKRDAVDPICRVPVITSLKEEQKL 168

KCTD10_human      153  IATSNKPAVKLLYNRSNNKYSYTSNSDDNMLKNIELFDKLSLRFNGRVLFIKDVIG 208
KCTD10_mouse      153  IATSNKPAVKLLYNRSNNKYSYTSNSDDNMLKNIELFDKLSLRFNGRVLFIKDVIG 208
Kctd10_zebrafish  153  IATANKPTVKLLYNRSNNKYSYTSNSDDNMLKNIELFDKLSLRFNGRVLFIKDVIG 208
kctd10_lamprey    169  ITTSNKPVVKLLYNRSNNKYSYTSNSDDNMLKNIELFDKLSLRFNGRVLFIKDVIG 224

KCTD10_human      209  DEICCWSFYGQGRKIAEVCCTSIVYATEKKQTKVEFPEARIYEETLNILLYEAQDG 264
KCTD10_mouse      209  DEICCWSFYGQGRKIAEVCCTSIVYATEKKQTKVEFPEARIYEETLNILLYEAQDG 264
Kctd10_zebrafish  209  DEICCWSFYGQGRKIAEVCCTSIVYATEKKQTKVEFPEARIYEETLNILLYESQDG 264
kctd10_lamprey    225  DEICCWSFYGQGRKIAEVCCTSIVYATEKKQTKVEFPEARIYEETLNILLYEPRDG 280

KCTD10_human      265  RG-PDNALLEATGGAAGRSHH-LDEDEERER--IERVRRIHIKRPDDRAHLHQ 313
KCTD10_mouse      265  RG-PDNALLEATGGAAGRSHH-LDEDEERERERIERVRRIHIKRPDDRAHLHQ 315
Kctd10_zebrafish  265  RG-PDNALLEATGGAAGRSHH-IDEDEERER--HDRVRRIHIKRPDDRTHHHQ 313
kctd10_lamprey    281  LGGPDKALLEATGGAAGRSHGPPDEDDDRDR--IDRVRRIHVKRLDDRSHQHQ 331
```

Figure S6. The alignment of the sequences of Kctd10 (encoded by the gene of *kctd10*) among human, mouse, zebrafish and lamprey. The target site of Cas9-gRNA is labeled with the red box.

Figure S7.

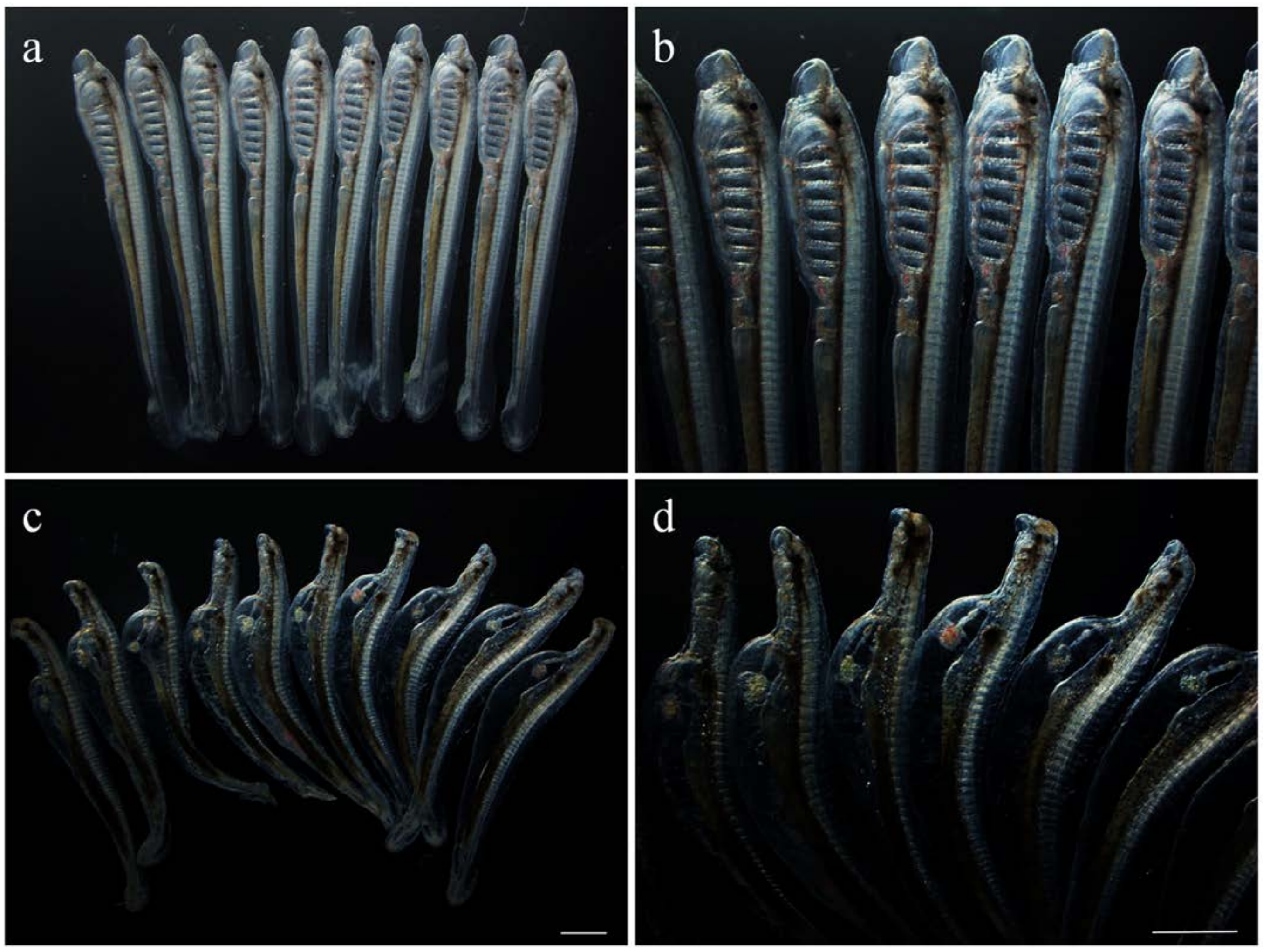

Figure S7. Injection of Cas9 and gRNA against *kctd10* resulting in heart malformations in lamprey larvae caused by biallelic disruption. Lateral views of control lamprey larvae (**a**, **b**) and *kctd10* gRNA injected larvae (**c**, **d**) at 21 dpf. The injected larvae showed severe pericardial edema. **d**, Enlarged view of **c** showed the marked heart malformations in the injected larvae. (Scale bars: 1mm).

Figure S8.

A

H1

```
Reference  ATCCTCAACTTCCTGCGCGACGGCGCCGTGCCCCTGCCGGAGTCTCGCACCGAGATCGAGGAGCTGCTCACAGAGGCCAAGTACTACC
Seq1:      ATCCTCAACTTCCTGCGCGACGGCGCCGTGCCCCTGCCGGAGTCTCGCACCGAG------GAGCTGCTCACAGAGGCCAAGTACTACC (-6bp)
Seq2:      ATCCTCAACTTCCTGCGCGACGGCGCCGTGCCCCTGCCGGAGTCTCGCACCGAGA------AGCTGCTCACAGAGGCCAAGTACTACC(-6bp)
Seq3:      ATCCTCAACTTCCTGCGCGACGGCGCCGTGCCCCTGCCGGAGTCTCGCACCGAG------GAGCTGCTCACAGAGGCCAAGTACTACC(-6bp)
Seq4:      ATCCTCAACTTCCTGCGCGACGGCGCCGTGCCCCTGCCGGAGTCTCGCACCGAG---------CTGCTCACAGAGGCCAAGTACTACC(-9bp)
Seq5:      ATCCTCAACTTCCTGCGCGACGGCGCCGTGCCCCTGCCGGAGTCTCGCACCGAG---------CTGCTCACAGAGGCCAAGTACTACC(-9bp)
Seq6:      ATCCTCAACTTCCTGCGCGACGGCGCCGTGCCCCTGCCGGAGTCTC------------------TGCTCACAGAGGCCAAGTACTACC(-18bp)
Seq7:      ATCCTCAACTTCCTGCGCGACGGCGCCGTGC------------------------------------TCACAGAGGCCAAGTACTACC(-36bp)
Seq8:      ATCCTCAACTTCCTGCGCGACGGCGCCGTGCCCCTGCCGGAGTCTCGCAC---------------------AGAGGCCAAGTACTACC(-21bp)
Seq9:      ATCCTCAA------G------------------------------------------GAGGAGCTGCTCACAGAGGCCAAGTACTACC(-48bp)
Seq10:     ATCCTCAACTTCCTGCGCGACGGCGCCGTGCCCCTGCCGGAGTCTC-----------------------ACAGAGGCCAAGTACTACC(-23bp)
Seq11:     ATCCTCAACTTCCTGCGCGACGGCGCCGTGCCCCTGCCGGAGTCTC-----------------------ACAGAGGCCAAGTACTACC(-23bp)
Seq12:     ATCCTCAACTTCCTGCGCGACGGCGCCGTGCCCCTGCCGGAGTCTC-----------------------ACAGAGGCCAAGTACTACC(-23bp)
Seq13:     ATCCTCAACTTCCTGCGCGACGGCGCCGTGC-----------------------------------------------------TACC(-53bp)
Seq14:     ATCCTCAACTTC-------------------------------------------------------TCACAGAGGCCAAGTACTACC(-55bp)
Seq15:     ----------------------------------------------------------AGGAGCTGCTCACAGAGGCCAAGTACTACC(-149bp)
Seq16:     ---------------------------------------------------------GAGGAGCTGCTCACAGAGGCCAAGTACTACC(-187bp)
Seq17:     ATCCTCAACTTCCTGCGCGACGGCGCCGTGCCCCTGCCGGAGTCTCGCACCGAGATCGAGGAGCTGCTCACAGAGGCCAAGTACTACC(wt)
Seq18:     ATCCTCAACTTCCTGCGCGACGGCGCCGTGCCCCTGCCGGAGTCTCGCACCGAGATCGAGGAGCTGCTCACAGAGGCCAAGTACTACC(wt)
Seq19:     ATCCTCAACTTCCTGCGCGACGGCGCCGTGCCCCTGCCGGAGTCTCGCACCGAGATCGAGGAGCTGCTCACAGAGGCCAAGTACTACC(wt)
Seq20:     ATCCTCAACTTCCTGCGCGACGGCGCCGTGCCCCTGCCGGAGTCTCGCACCGAGATCGAGGAGCTGCTCACAGAGGCCAAGTACTACC(wt)
```

H2

```
Reference  ATCCTCAACTTCCTGCGCGACGGCGCCGTGCCCCTGCCGGAGTCTCGCACCGAGATCGAGGAGCTGCTCACAGAGGCCAAGTACTACC
Seq1:      ATCCTCAACTTCCTGCGCGACGGCGCCGTGCCCCTGCCGGAGTCTCGCACCGAG------GAGCTGCTCACAGAGGCCAAGTACTACC(-6bp)
Seq2:      ATCCTCAACTTCCTGCGCGACGGCGCCGTGCCCCTGCCGGAGTCTCGCACCGAG------GAGCTGCTCACAGAGGCCAAGTACTACC(-6bp)
Seq3:      ATCCTCAACTTCCTGCGCGACGGCGCCGTGCCCCTGCCGGAGTCTCGCACCGAG------GAGCTGCTCACAGAGGCCAAGTACTACC(-6bp)
Seq4:      ATCCTCAACTTCCTGCGCGACGGCGCCGTGCCCCTGCCGGAGTCTCGCA------TCGAGGAGCTGCTCACAGAGGCCAAGTACTACC(-6bp)
Seq5:      ATCCTCAACTTCCTGCGCGACGGCGCCGTGCCCCTGCCGGAGTCTCGCACCGAG------GAGCTGCTCACAGAGGCCAAGTACTACC(-6bp)
Seq6:      ATCCTCAACTTCATGCGCGACGGCGCCATCCCCCAGCCGGAGTCTGGCAC------CGAGGAGCTGCTCACAGAGGCCAAGTACTACC(-6bp)
Seq7:      ATCCTCAACTTCCTGCACGACGGCGCCGTGCCCCTGCCGGAGTCTCGCACCGAG------GAGCTGCTCACAGAGGCCAAGTACTACC(-6bp)
Seq8:      ATCCTCAACTTCCTGCGCGACGGCGCCGTGCCCCTGCCGGAGT---------------------TGCTCACAGAGGCCAAGTACTACC(-21bp)
Seq9:      ATCCTC---------------------------------------------------GAGGAGCTGCTCACAGAGGCCAAGTACTACC(-51bp)
Seq10:     ATCCTCAA------------------------------------------------------------------GGCCAAGTACTACC(-66bp)
Seq11:     ATCCTCAACTTCCTGCGCGACGGCGCCGTGCCCCTGCCGGAGTCTCGCACCGAGA--GAGGAGCTGCTCACAGAGGCCAAGTACTACC(-2bp)
Seq12:     ATCCTCAACTTCCTGCGCGACGGCGCCGTGCCCCTGCCGGAGTCTCGCAC-----T-GAGGAGCTGCTCACAGAGGCCAAGTACTACC(-5bp)
Seq13:     ATCCTCAACTTCCTGCGCGACGGCGCCGTGCCCCTGCCGGAGTCTCG-----------AGGAGCTGCTCACAGAGGCCAAGTACTACC(-11bp)
Seq14:     ATCCTCAACTTCCTGCGCGACGGCGCCGTGCCCCTGCCGGAGTCTCG-----------AGGAGCTGCTCACAGAGGCCAAGTACTACC(-11bp)
Seq15:     ATCCTCAACTTCCTGCGCGACGGCGCCGTGCCCCTGCCG-------------------AGGAGCTGCTCACAGAGGCCAAGTACTACC (-19bp)
Seq16:     ATCCTCAACTTCCTGCGCGACGGCGCCGTGCCCCTGCCGGAGTCT--------------------GCTCACAGAGGCCAAGTACTACC(-20bp)
Seq17:     ----------------------------------------------------------------TGCTCACAGAGGCCAAGTACTACC(-67bp)
Seq18:     ---------------------------------------------------------GAGGAGCTGCTCACAGAGGCCAAGTACTACC(-227bp)
Seq19:     ATCCTCAACTTCCTGCGCGACGGCGCCGTGCCCCTGCCGGAGTCTCGCACCGAGATCGAGGAGCTGCTCACAGAGGCCAAGTACTACC(wt)
```

H3

```
Reference  ATCCTCAACTTCCTGCGCGACGGCGCCGTGCCCCTGCCGGAGTCTCGCACCGAGATCGAGGAGCTGCTCACAGAGGCCAAGTACTACC
Seq1:      ATCCTCAACTTCCTGCGCGACGGCGCCGTGCCCCTGCCGGAGTCTCGCACCGAG------GAGCTGCTCACAGAGGCCAAGTACTACC (-6bp)
Seq2:      ATCCTCAACTTCCTGCGCGACGGCGCCGTGCCCCTGCCGGAGTCTCGCACCGAG------GAGCTGCTCACAGAGGCCAAGTACTACC (-6bp)
Seq3:      ATCCTCAACTTCCTGCGCGACGGCGCCGTGCCCCTGCCGGAGTCTCGCACCGAG------GAGCTGCTCACAGAGGCCAAGTACTACC (-6bp)
Seq4:      ATCCTCAACTTCCTGCGCGACGGCGCCGTGCCCCTGCCGGAGTCTCGCACCGAG------GAGCTGCTCACAGAGGCCAAGTACTACC (-6bp)
Seq5:      ATCCTCAACTTCCTGCGCGACGGCGCCGTGCCCCTGCCGGAGTCTCGCACCGAG------GAGCTGCTCACAGAGGCCAAGTACTACC (-6bp)
Seq6:      ATCCTCAACTTCCTGCGCGACGGCGCCGTGCCCCTGCCGGAGTCTCGCACCGAG------GAGCTGCTCACAGAGGCCAAGTACTACC(-6bp)
Seq7:      ATCCTCAACTTCCTGCGCGACGGCGCCGTGCCCCTGCCGGAGTCTCGCACCGAG------GAGCTGCTCACAGAGGCCAAGTACTACC(-6bp)
Seq8:      ATCCTCAACTTCCTGCGCGACGGCGCCGTGCCCCTGCCGGAGTCTCGCACCGAGCAGCTCCTCGAGGAGCTGCTCACAGAGGCCAAGTA (+7, -1bp)
Seq9:      ATCCTCAACTTCCTGCGCGACGGCGCCGTGCCCCTGCCGGAGTCT-----------CGAGGAGCTGCTCACAGAGGCCAAGTACTACC(-11bp)
Seq10:     ATCCTCAACTTCCTGCGCGACGGCGCC-------------------------------------------------------------(-97bp)
Seq11:     ATCCTCAACTTCCTGCGCGACGGCGCCGTGCCCCTGCCGGAGTCTCGCACCGAGAATCGAGGAGCTGCTCACAGAGGCCAAGTACTACC (+1bp)
Seq12:     ATCCTCAACTTCCTGCGCGACGGCGCCGTGCCCCTGCCGGAGTCTCGCACCGAGAATCGAGGAGCTGCTCACAGAGGCCAAGTACTACC (+1bp)
Seq13:     ATCCTCAACTTCCTGCGCGACGGCGCCGCGCCCCTGCCGGAGTCTCGCACCGAGAGTCGAGGAGCTGCTCACAGAGGCCAAGTACTACC (+1bp)
Seq14:     ATCCTCAACTTCCTGCGCGACGGCGCCGTGCCCCTGCCGGAGTCTCGCACCGAGATCCAGGAGCTGCTCACAGAGGCCAAGTACTACC(wt)
```

```
Seq15:     ATCCTCAACTTCCTGCGCGACGGCGCCGTGCCCCTGCCGGAGTCTCGCACCGAGATCGAGGAGCTGCTCACAGAGGCCAAGTACTACC(wt)
Seq16:     ATCCTCAACTTCCTGCGCGACGGCGCCGTGCCCCTGCCGGAGTCTCGCACCGAGATCGAGGAGCTGCTCACAGAGGCCAAGTACTACC(wt)
Seq17:     ATCCTCAACTTCCTGCGCGACGGCGCCGTGCCCCTGCCGGAGTCTCGCACCGAGATCGAGGAGCTGCTCACAGAGGCCAAGTACTACC(wt)
```

B

W1

```
Reference  ATCCTCAACTTCCTGCGCGACGGCGCCGTGCCCCTGCCGGAGTCTCGCACCGAGATCGAGGAGCTGCTCACAGAGGCCAAGTACTACC
Seq1:      ATCCTCAACTTCGTGCGCGCCGGCGCCGTGCCCCAGCCGGAGTCTCGCACCGAG------GAGCTGCTCACAGAGGCCAGGTACTACC(-6bp)
Seq2:      ATCCTCAACTTCCTGCGCGACGGCGCCGTGCCCCTGCCGGAGTCTCGCACCGAG------GAGCTGCTCACAGAGGCCAAGTACTACC(-6bp)
Seq3:      ATCCTCAACTTCCTGCGCGACGGCGCCGTGCCCCTGCCGGAGTCTCGCACCGAG------GAGCTGCTCACAGAGGCCAAGTACTACC(-6bp)
Seq4:      ATCCTCAACTTCCTGCGCGACGGCGCCGTGCCCCTGCCGGAGTCTCGCACCGAGATCGAGGAGCTGCTC------GCCAAGTACTACC(-6bp)
Seq5:      ATCCTCAACTTCCTGCGCGACGGCGCCGTGCCCCTGCCGGAGTCTCGCACCGAGAT---------GCTCACAGAGGCCAAGTACTACC(-9bp)
Seq6:      ATCCTCAACTTCCTGCGCGACGGCGCCGTGCCCCTGCCGGAG------------------GAGCTGCTCACAGAGGCCAAGTACTACC(-18bp)
Seq7:      ATCCTCAACTTCCTGCGC--------------------------------------------------------ACAGAGGCCAAGTACTACC(-51bp)
Seq8:      ATCCTCAACTTCCTGCGCGACGGCGCCGTGCCCCTGCCGGAGTCTCGCACCGAGATCGAGGAGCTGCTC-------------------(-19bp)
Seq9:      ATCCTCAACTTCCTGCGCGACGGCGCCGTGCCCCTGCCGGAGTCTCGC-------------------TCACAGAGGCCAAGTACTACC(-19bp)
Seq10:     ATCCTCAACTTCCTGCGCGACGGCGCCGTGCCCCTGCCGGAGTCTCGCAC-------------------------------------TACC(-34bp)
Seq11:     ATCCTCAACTTCCTGCGCGACGGCGCCGTGCCCCTGCCGGAGTCTCGCACCGAGATCGAGGAGCTGCTCACAGAGGCCAAGTACTACC(wt)
Seq12:     ATCCTCAACTTCCTGCGCGACGGCGCCGTGCCCCTGCCGGAGTCTCGCACCGAGATCGAGGAGCTGCTCACAGAGGCCAAGTACTACC(wt)
Seq13:     ATCCTCAACTTCCTGCGCGACGGCGCCGTGCCCCTGCCGGAGTCTCGCACCGAGATCGAGGAGCTGCTCACAGAGGCCAAGTACTACC(wt)
Seq14:     ATCCTCAACTTCCTGCGCGACGGCGCCGTGCCCCTGCCGGAGTCTCGCACCGAGATCGAGGAGCTGCTCACAGAGGCCAAGTAGTACC(wt)
Seq15:     ATCCTCAACTTCCTGCGCGACGGCGCCGTGCCCCTGCCGGAGTCTCGCACCGAGATCGAGGAGCTGCTCACAGAGGCCAAGTACTACC(wt)
Seq16:     ATCCTCAACTTCCTGCACGACGGCGCCGTGCCCCTGCCGGAGTCTCGCACCGAGATCGAGGAGCTGCTCACAGAGGCCAAGTACTACC(wt)
Seq17:     ATCCTCAACTTCATGCGCGACGGCGCCGTGCCCCTGCCGGAGTCTCGCACCGAGATCGAGGAGCTGCTCACAGAGGCCAAGTACTACC(wt)
Seq18:     ATCCTCAACTTCCTGCGCGACGGCGCCGTGCCCCTGCCGGAGTCTCGCACCGAGATCGAGGAGCTGCTCACAGAGGCCAAGTACTACC(wt)
```

W2

```
Reference  ATCCTCAACTTCCTGCGCGACGGCGCCGTGCCCCTGCCGGAGTCTCGCACCGAGATCGAGGAGCTGCTCACAGAGGCCAAGTACTACC
Seq1:      ATCCTCAACTTCCTGCGCGACGGCGCCGTGCCCCTGCCGGAGTCTCGCAC------CGAGGAGCTGCTCACAGAGGCCAAGTACTACC(-6bp)
Seq2:      ATCCTCAACTTCCTGCGCGACGGCGCCGTGCCCCTGCCGGAGTCTCGCACCGAG------GAGCTGCTCACAGAGGCCAAGTACTACC(-6bp)
Seq3:      ATCCTCAACTTCCTGCGCGACGGCGCCGTGCCCCTGCCGGAGTCTCGCACCGAG------GAGCTGCTCACAGAGGCCAAGTACTACC(-6bp)
Seq4:      ATCCTCAACTTCCTGCGCGACGGCGCCGTGCCCCTGCCGGAGTCTCGCA------TCGAGGAGCTGCTCACAGAGGCCAAGTACTACC(-6bp)
Seq5:      ---------------------------------------------------------------GAGGAGCTGCTCACAGAGGCCAAGTACTACC(-81bp)
Seq6:      ATCCTCAACTTCCTGCGCGACGGCGCCGTGCCCCTGCCGGAGTCTCGCA----------GGAGCTGCTCACAGAGGCCAAGTACTACC(-10bp)
Seq7:      ATCCTCAACTTCGTGCGGGACGGCGCC-----------------------------CGAGGAGCTGCTCACAGAGGCCAAGTACTACC(-29bp)
Seq8:      ATCCTCAACTTCCTGCGCGACGGCGCCGT----------------------------------------------------------ACTACC(-53bp)
Seq9:      -----------------------------------------------------------------GGAGCTGCTCACAGAGGCCAAGTACTACC(-71,+1bp)
Seq10:     ATCCTCAACTTCCTGCGCGACGGCGCCGTGCCCCTGCCGGAGTCTCGCACCGAGAGACTCCGGCAGAGGAGCTGCTCACAGAGGCCAA(-2,+10bp)
Seq11:     ATCCTCAACTTCCTGCGCGACGGCGCCGTGCCCCTGCCGGAGTCTCGCACCGAGAGACTCCGGCAGAGGAGCTGCTCACAGAGGCCAA(-2,+10bp)
Seq12:     ATCCTCAACTTCCTGCGCGACGGCGCCGTGCCCCTGCCGGAGTCTCGCACCGAGATCGAGGAGCTGCTCACAGAGGCCAAGTACTACC(wt)
Seq13:     ATCCTCAACTTCCTGCGCGACGGCGCCGTGCCCCTGCCGGAGTCTCGCACCGAGATCGAGGAGCTGCTCACAGAGGCCAAGTACTACC(wt)
Seq14:     ATCCTCAACTTCCTGCGCGACGGCGCCGTGCCCCTGCCGGAGTCTCGCACCGAGATCGAGGAGCTGCTCACAGAGGCCAAGTACTACC(wt)
Seq15:     ATCCTCAACTTCCTGCGCGACGGCGCCGTGCCCCTGCCGGAGTCTCGCACCGAGATCGAGGAGCTGCTCACAGAGGCCAAGTACTACC(wt)
Seq16:     ATCCTCAACTTCCTGCGCGACGGCGCCGTGCCCCTGCCGGAGTCTCGCACCGAGATCGAGGAGCTGCTCACAGAGGCCAAGTACTACC(wt)
Seq17:     ATCCTCAACTTCCTGCGCGACGGCGCCGTGCCCCTGCCGGAGTCTCGCACCGAGATCGAGGAGCTGCTCACAGAGGCCAAGTACTACC(wt)
Seq18:     ATCCTCAACTTCCTGCGCGACGGCGCCGTGCCCCTGCCGGAGTCTCGCACCGAGATCGAGGAGCTGCTCACAGAGGCCAAGTACTACC(wt)
Seq19:     ATCCTCAACTTCCTGCGCGACGGCGCCGTGCCCCTGCCGGAGTCTCGCACCGAGACCGAGGAGCTGCTCACAGAGGCCAAGTACTACC(wt)
Seq20:     ATCCTCAACTTCCTGCGCGACGGCGCCGTGCCCCTGCCGGAGTCTCGCACCGAGATCGAGGAGCTGCTCACAGAGGCCAAGTACTACC(wt)
```

W3

```
Reference  ATCCTCAACTTCCTGCGCGACGGCGCCGTGCCCCTGCCGGAGTCTCGCACCGAGATCGAGGAGCTGCTCACAGAGGCCAAGTACTACC
Seq1:      ATCCTCAACTTCCTGCGCGACGGCGCCGTGCCCCTGCCGGAGTCTCGCACCGAG------GAGCTGCTCACTGAGGCCAAGTACTACC(-6bp)
Seq2:      ATCCTCAACTTCCTGCGCGACGGCGCCGTGCCCCTGCCGGAGTCTCGCACCGAG---------CTGCTCACAGAGGCCAAGTACTACC(-9bp)
Seq3:      ATCCTCAACTTCCTGCGCGACGGCGCCGTGCCCCTGCCGGAGT------------TCGAGGAGCTGCTCACAGAGGCCAAGTACTACC(-12bp)
Seq4:      ATCCTCAACTTCCTGCGCGACGGCGCCGTGCCCCT---------------------CGAGGAGCTGCTCACAGAGGCCAAGTACTACC（-21bp）
Seq5:      ATCCTCAACTTCCTGCGCGACGGCGCCGTGCCCCTGCCGGAGTCTCG-----------AGGAGCTGCTCACAGAGGCCAAGTACTACC(-11bp)
Seq6:      ATCCTCAACTTCCTGCGCGACGGCGCCGTGCCCCTGCCGGAGTCTCGCACCGAGAGTCGAGGAGCTGCTCACAGAGGCCAAGTACTACC(+1bp)
Seq7:      ATCCTCAACTTCCTGCGCGACGGCGCCGTGCCCCTGCCGGAGTCTCGCACCGAGATCGAGGAGCTGCTCACAGAGGCCAAGTACTACC(wt)
Seq8:      ATCCTCAACTTCCTGCGCGACGGCGCCGTGCCCCTGCCGGAGTCTCGCACCGAGATCGAGGAGCTGCTCACAGAGGCCAAGTACTACC(wt)
Seq9:      ATCCTCAACTTCCTGCGCGACGGCGCCGTGCCCCTGCCGGAGTCTCGCACCGAGATCGAGGAGCTGCTCACAGAGGCCAAGTACTACC(wt)
Seq10:     ATCCTCAACTTCCTGCGCGACGGCGCCGTGCCCCTGCCGGAGTCTCGCACCGAGATCGAGGAGCTGCTCACAGAGGCCAAGTACTACC(wt)
Seq11:     ATCCTCAACTTCCTGCGCGACGGCGCCGTGCCCCTGCCGGAGTCTCGCACCGAGATCGAGGAGCTGCTCACAGAGGCCAAGTACTACC(wt)
Seq12:     ATCCTCAACTTCCTGCGCGACGGCGCCGTGCCCCTGCCGGAGTCTCGCACCGAGATCGAGGAGCTGCTCACAGAGGCCAAGTACTACC(wt)
```

```
Seq13:      ATCCTCAACTTCCTGCGCGACGGCGCCGTGCCCCTGCCGGAGTCTCGCACCGAGATCGAGGAGCTGCTCACAGAGGCCAAGTACTACC(wt)
Seq14:      ATCCTCAACTTCCTGCGCGACGGCGCCGTGCCCCTGCCGGAGTCTCGCACCGAGATCGAGGAGCTGCTCACAGAGGCCAAGTACTACC(wt)
Seq15:      ATCCTCAACTTCCTGCGCGACGGCGCCGTGCCCCTGCCGGAGTCTCGCACCGAGATCGAGGAGCTGCTCACAGAGGCCAAGTACTACC(wt)
Seq16:      ATCCTCAACTTCCTGCGCGACGGCGCCGTGCCCCTGCCGGAGTCTCGCACCGAGATCGAGGAGCTGCTCACAGAGGCCAAGTACTACC(wt)
Seq17:      ATCCTCAACTTCCTGCGCGACGGCGCCGTGCCCCTGCCGGAGTCTCGCACCGAGATCGAGGAGCTGCTCACAGAGGCCAAGTACTACC(wt)
Seq18:      ATCCTCAACTTCCTGCGCGACGGCGCCGTGCCCCTGCCGGAGTCTCGCACCGAGATCGAGGAGCTGCTCACAGAGGCCAAGTACTACC(wt)
Seq19:      ATCCTCAACTTCCTGCGCGACGGCGCCGTGCCCCTGCCGGAGTCTCGCACCGAGATCGAGGAGCTGCTCACAGAGGCCAAGTACTACC(wt)
```

Figure S8. The sequences of the target region of *kctd10* in the lamprey larvae injected with Cas9 and *kctd10* gRNA. **A**, The sequences of the target region of *kctd10* in the injected lamprey larvae with heart malformations; **B**, The sequences of the target region of *kctd10* in the injected lamprey larvae with normal phenotypes. The target sites are labeled with box, the PAM sequences are highlighted with yellow and insertions are highlighted with red.

Figure S9.

A

wee1 No.1

```
Reference    TCCCGACCGAGAGGCCTCAAGCCTCGGCCTCGCCTTGACACCTCCGCCGGGCGCCTCGCACCGAAGCCGGCGGGTCGTCCAGGCCACG
Seq1:        TCCCGACCGAGAGGCCTCAAGCCTCGGCCTCGCCT------------CGGGCGCCTCGCACCGAAGCCGGCGGGTCGTCCAGGCCACG（-12bp）
Seq2:        TCCCGACCGAGAGGCCTCAAGCCTCGGCCTCGCCT---------------------CGCACCGAAGCCGGCGGGTCGTCCAGGCCACG（-21bp）
Seq3:        TCCCGACCGAGAGGCCTCAAGCCTCGGCC---------------------------------------------------AGGCCACG（-51bp）
Seq4:        TCCCGACCGAGAGGCCTCAAGCCTCGGCCTCGCC-----ACCTCCGCCGGGCGCCTCGCACCGAGGCCGGCGGGTCGTCCAGGCCACG（-5bp）
Seq5:        TCCCGACCGAGAGGCCTCAAGCCTCGGCCTC--------ACCTCCGCCGGGCGCCTCGCACCGAAGCCGGCGGGTCGTCCAGGCCACG（-8bp）
Seq6:        TCCCGACCGAGAGGCCTCAAGCCTCGGCCTCGCC--------------GGGCGCCTCGCACCGAAGCCGGCGGGTCGTCCAGGCCACG（-14bp）
Seq7:        TCCCGACCGAGAGGCCTCAAGCCTCGGCCTCGCC--------------GGGCGCCTCGCACCGAAGCCGGCGGGTCGTCCAGGCCACG（-14bp）
Seq8:        TCCCGACCGAGAGGCCTCAAGCCTCGGCCTCGCC--------------GGGCGCCTCGCACCGAAGCCGGCGGGTCGTCCAGGCCACG（-14bp）
Seq9:        TCCCGACCGAGAGGCCTC--------------------------CGCCGGGCGCCTCGCACCGAAGCCGGCGGGTCGTCCAGGCCACG（-26bp）
Seq10:       TCCCGACCGAGAGGCCTCAAGCCTCGGCCTCGCCTTGA-------G-------------------GCCGGCGGGTCGTCCAGGCCACG（-26bp）
Seq11:       TCCCGACCGAGAGGCCTCAAGCCTCGGCCTCGC--------------------------ACCGAAGCCGGTGGGTCGTCCAGGCCACG（-26bp）
Seq12:       TCCCGACCGAGAGGCCTCAAGCCTCGGCCTCGC--------------------------ACCGAAGCCGGCGGGTCGTCCAGGCCACG（-26bp）
Seq13:       TCCCGACCGAGAGGCCTC--------------------------CGCCGGGCGCCTCGCACCGAAGCCGGCGGGTCGTCCAGGCCACG（-26bp）
Seq14:       TCCCGACCGAGAGGCCTCAAGCCTCG--------------------------------CACCGAAGCCGGCGGGTCGTCCAGGCCACG（-32bp）
Seq15:       TCCCGACC----------------------------------TCCGCCGGGCGCCTCGCACCGAAGCCGGCGGGTCGTCCAGGCCACG（-34bp）
Seq16:       TCCCGACC----------------------------------TCCGCCGGGCGCCTCGCACCGAAGCCGGCGGGTCGTCCAGGCCACG（-34bp）
Seq17:       TCCCGACCGAGAGGCCTCAAGCCTCGGCCTCGGCCT---CACCTCCGCCGGGCGCCTCGCACCGAAGCCGGCGGGTCGTCCAGGCCAC（+1，-3bp）
Seq18:       TCCCGACCGAGAGGCCTCAAGCCTCGGCCTCGGCCT---CACCTCCGCCGGGCGCCTCGCACCGAAGCCGGCGGGTCGTCCAGGCCAC（+1，-3bp）
Seq19:       TCCCGACCGAGAGGCCTCAAGCCTCGGCCTCGCCTTGAGGCGAG-GCCGGGCGCCTCGCACCGAAGCCGGCGGGTCGTCCAGGCCACG（+6，-7bp）
Seq20:       TCCCGACCGAGAGGCCTCAAGCCTCGGCCTCGCCTTGACACCTCCGCCGGGCGCCTCGCACCGAAGCCGGCGGGTCGTCCAGGCCACG(wt)
```

wee1 No.2

```
Reference    TCCCGACCGAGAGGCCTCAAGCCTCGGCCTCGCCTTGACACCTCCGCCGGGCGCCTCGCACCGAAGCCGGCGGGTCGTCCAGGCCACG
Seq1:        TCCCGACCGAGAGGCCTCAAGCCTCGGCCT---------ACCTCCGCCGGGCGCCTTGCTCGGTAGCCGGCTGGTCGTCCAGGCCCCG（-9bp）
Seq2:        TCCCGACCGAGAGGCCTCAAGCCTCGGCCTCGCCT---------------------CGCACCGAAGCCGGCGGGTCGTCCAGGCCACG（-21bp）
Seq3:        TCCCGACCGAGAGGCCTC---------------------------------------GCACCGAAGCCGGCGGGTCGTCCAGGCCACG（-39bp）
Seq4:        TCCCGACCGAGAGGCCTC---------------------------------------GCACCGAAGCCGGCGGGTCGTCCAGGCCACG（-39bp）
Seq5:        TCCCGGCCGAGAGGCCTCAAGCCTCGGCCTCGCC-----ACCTCCGCCGGGCGCCTCGCACCGAAGCCGGCGGGTCGTCCAGGCCACG（-5bp）
Seq6:        TCCCGACCGAGAGGCCTCAAGCCTCGGCCTCGCC-----ACCTCCGCCGGGCGCCTCGCACCGAAGCCGGCGGGTCGTCCAGGCCACG（-5bp）
Seq7:        TCCCGACCGAGAGGCCTCAAGCCTCGGCCTCGCC-----ACCTCCGCCGGGCGCCTCGCACCGAAGCCGGCGGGTCGTCCAGGCCACG（-5bp）
Seq8:        TCCCGACCGAGAGGCCTCAAGCCTCGGCCTCGCCT--------CCGCCGGGCGCCTCGCACCGAAGCCGGCGGGTCGTCCAGGCCACG（-8bp）
Seq9:        TCCCGACCGAGAGGCCTCAAGCCTCGGCCTCGCCT--------CCGCCGGGCGCCTCGCACCGAAGCCGGCGGGTCGTCCAGGCCACG（-8bp）
Seq10:       TCCCGACCGAGAGGCCTCAAGCCTCGGCCTCGCCT--------CCGCCGGGCGCCTCGCACCGAAGCCGGCGGGTCGTCCAGGCCACG（-8bp）
Seq11:       TCCCGACCGAGAGGCCTCAAGCCTCGGCCTCGCCT--------CCGCCGGGCGCCTCGCACCGAAGCCGGCGGGTCGTCCAGGCCACG（-8bp）
Seq12:       TCCCGACCGAGAGGCCTCAAGCCTCGGCCTCGCC--------------GGGCGCCTCGCACCGAAGCCGGCGGGTCGTCCAGGCCACG（-14bp）
Seq13:       TCCCGACCGAGGGGCCTCAAG-------------------CCTCCGCCGGGCGCCTCGCACCGAAGCCGGCGGGTCGTCCAGGCCACG（-19bp）
Seq14:       TCCCGACCGAGAGGCCTCAAGCC-------------------------GGGCGCCTCGCACCGAAGCCGGCGGGTCGTCCAGGCCACG（-25bp）
Seq15:       TCCCGACCGAGAGGCCTCAAGCCTCGGCCTCGC--------------------------ACCGAAGCCGGGGGGTCGTCCAGGCCACG（-26bp）
Seq16:       TCCCGACCGAGAGGCCTCAAGCCTCGGCCTCGCCTTGA--------------------------AGCCGGCGGGTCGTCCAGGCCACG（-26bp）
Seq17:       TCCCGACCGAGAGGCCTCAAGCCTCGGCCTCGC--------------------------ACCGAAGCCGGCGGGTCGTCCAGGCCACG（-26bp）
Seq18:       TCCCGACCGAGAGGCCTCAAGCCTCGGCCTCGC--------------------------ACCGAAGCCGGCGGGTCGTCCAGGCCACG（-26bp）
Seq19:       TCCCGACCGAGAGGCC--------------------------------GGGCGCCTCGCACCGAAGCCGGCGGGTCGTCCAGGCCACG（-32bp）
Seq20:       TCCCGACCGAGAGGCCTCAAGCCTCGGCCTCGC-------------------------------------------------------（-61bp）
```

B

soxe2 No.1

```
Reference    GAGGCACCGACGACCCCTTCTCGGAGAGCATCCAGGCGGCCGTGAGTCAAGTGCTGGACGGCTACGACTGGTCGCTGTTGCCCGTGCCC
Seq1:        GAGGCACCGACGACCCCTTCTCGGAGAGCATCCAGGCGGCCGTGAGTCA----C-GGCCGCCTG-GACTGGTCGCTGTTGCCCGTGCCC（-6bp）
Seq2:        GAGGCACCGACGACCCCTTCTCGGAGAGCATCCAGGCGGCCGT----------------------------------------------(-60bp)
Seq3:        GAGGCACCGACGACCCCTTCTCGGAGAGCATCCAGGCGGCCGTGAG----------GACGGCTACGACTGGTCGCTGTTGCCCGTGCCC（-10bp）
Seq4:        GAGGCACCGACGACCCCTTCTCGGAGAGCATCCAGGCGGC----------------------TACGACTGGTCGCTGTTGCCCGTGCCC（-22bp）
Seq5:        GAGGCACCGACGACCCCTTCTCGGAGAGCATCCAGGCGGC----------------------TACGACTGGTCGCTGTTGCCCGTGCCC（-22bp）
Seq6:        GAGGCACCGACGACCCCTTCTCGGAGAGCATCCAGGCGGC----------------------TACGACTGGTCGCTGTTGCCCGTGCCC（-22bp）
Seq7:        GAGGCACCGACGACCCCTTCTCGGAGAGCATCCAGGCGGC----------------------TACGACTGGTCGCTGTTGCCCGTGCCC（-22bp）
Seq8:        GAGGCACCGACGACCCCTTCTCGGAGAGCATCCAGGCGGC----------------------TACGACTGGTCGCTGTTGCCCGTGCCC（-22bp）
Seq9:        GAGGCACCGACGACCCCTTCTCGGAGAGCATCCAGGCGGC----------------------TACGACTGGTCGCTGTTGCCCGTGCCC（-22bp）
Seq10:       GAGGCACCGACGACCCCTTCTCGGAGAGCATCCAGGCGGC----------------------TACGACTGGTCGCTGTTGCCCGTGCCC（-22bp）
```

Seq11: GAGGCACCGACGACCCCTTCTCGGAGAGCATCCAGGCGGC----------------------TACGACTGGTCGCTGTTGCCCGTGCCC（-22bp）

Seq12: GAGGCACCGACGACCCCTTCTCGGAGAGCATCCAGGCGGC----------------------TACGACTGGTCGCTGTTGCCCGTGCCC（-22bp）

Seq13: GAGGCACCGACGACCCCTTCTCGGAGAGCATCCAGGCGGC----------------------TACGACTGGTCGCTGTTGCCCGTGCCC（-22bp）

Seq14: GAGGCACCGACGACCCCTTCTCGGAGAGCATCCAGGCGGC----------------------TACGACTGGTCGCTGTTGCCCGTGCCC（-22bp）

Seq15: GAGGCACCGACGACCCCTTCTCGGAGAGCATCCAGGCGGC----------------------------TGGTCGCTGTTGCCCGTGCCC（-28bp）

Seq16: GAGGCACCGACGA--CTGGACGGCTACGACTGGTCGCTGTTGCCCGTGCCC（-40bp）

Seq17: GAGGCACCGACGA--CTGGACGGCTACGACTGGTCGCTGTTGCCCGTGCCC（-40bp）

Seq18: GAGGCACCGACGACCCCTTCTCG---TGCCC（-61bp）

Seq19: GAGGCACCGACGACCCCTTCTCG---TGCCC（-61bp）

soxe2 No.2

Reference GAGGCACCGACGACCCCTTCTCGGAGAGCATCCAGGCGGCCGTGAGTCAAGTGCTGGACGGCTACGACTGGTCGCTGTTGCCCGTGC

Seq1: GAGGCACCGACGACCCCTTCTCGGAGAGCATCCAGGCGGCCGTG---------CTGGACGGCTACGACTGGTCGCTGTTGCCCGTGC（-9bp）

Seq2: GAGGCACCGACGACCCCTTCTCGGAGAGCATCCAGGCGGCCGTG-GACGGCTAC-—GACGGCTACGACTGGTCGCTGTTGCCCGTGC(-12,+9bp)

Seq3: GAGGCACCGACGACCCCTTCTCGGAGAGCATCCAGGCGGCCGTG-GACGGCTAC-—GACGGCTACGACTGGTCGCTGTTGCCCGTGC(-12,+9)

Seq4: GAGGCACCGACGACCCCTTCTCGGAGAGCATCCAGGCGGCCGTG-GACGGCTAC-—GACGGCTACGACTGGTCGCTGTTGCCCGTGC(-12,+9)

Seq5: GAGGCACCGACGACCCCTTCTCGGAGAGCATCCAGGCGGCCGTG-GACGGCTAC-—GACGGCTACGACTGGTCGCTGTTGCCCGTGC(-12,+9)

Seq6: GAGGCACCGACGACCCCTTCTCGGAGAGCATCCAGGCGGCCGTG------------GACGGCTACGACTGGTCGCTGTTGCCCGTGC（-12bp）

Seq7: GAGGCACCGACGACCCCTTCTCGGAGAGCA------------------------------------ACTGGTCGCTGTTGCCCGTGC（-36bp）

Seq8: GAGGCACCGACGACCCCTTCTCGGAGAGCA------------------------------------ACTGGTCGCTGTTGCCCGTGC（-36bp）

Seq9: ---GCTGGACGGCTACGACTGGTCGCTGTTGCCCGTGC(-66bp)

Seq10: ---GCTGGACGGCTACGACTGGTCGCTGC（-66bp）

Seq11: ---GCTGGACGGCTACGACTGGTCGCTGC（-66bp）

Seq12: ---GCTGGACGGCTACGACTGGTCGCTGC(-66bp)

Seq13: GAGGCACCGACGACCCCTTCTCGGAGAGCATCCAGGCGGCCGTGA-------------CGGCTACGACTGGTCGCTGTTGCCCGTGC（-13bp）

Seq14: GAGGCACCGACGACCCCTTCTCGGAGAGCATCCAGG-----------------------------------TCGCTGTTGCCCGTGC（-35bp）

Seq15: GAGGCACCGACGACCCCTTCTCGGAGAGCATCCAGG-----------------------------------TCGCTGTTGCCCGTGC（-35bp）

Seq16: GAGGCACCGACGACCCCTTCTCGGAGAGCATCCAGG-----------------------------------TCGCTGTTGCCCGTGC（-35bp）

Seq17: GAGGCACCGACGACCCCCTCTCGGAGAGCATCCAGG-----------------------------------TCGCTGTTGCCCGTGC（-35bp）

Seq18: GAGGCGCCGACGACCCCTTCTCGGAGAGCATCCAGGCGGCCGTGAGTCAAGTGCTGGACGGCTACGACTGGTCGCTGTTGCCCGTGC

C

wnt7b No.1

Reference GTACGAGCACACGGTGCACGTGGAGCCGGTGAGCGGCAGCCGTTACATGCGGCCGATGTTCCTCAAGGTGAAGAAGTCGCGCTCGTAC

Seq1: GTACGAGCACACGGTGCACGTGGAGCCGGTGAGCGGCAGCC------TGCGGCCGATGTTCCTCAAGGTGAAGAAGTCGCGCTCGTAC（-6bp）

Seq2: GTACGAGCACACGGT---CCTCAAGGTGAAGAAGTCGCGCTCGTAC（-45bp）

Seq3: GTACGAGCACACGGTGCACGTG--AAGAAGTCGCGCTCGTAC（-48bp）

Seq4: GTACGAGCACACGGTGCACGTGGAGCCGGTGAGCGGCAGC----------GGCCGATGTTCCTCAAGGTGAAGAAGTCGCGCTCGTAC（-10bp）

Seq5: GTACGAGCACACGGTGCACGTGGAGCCGGTGAGCGGCAGCCG-------------ATGTTCCTCAAGGTGAAGAAGTCGCGCTCGTAC（-13bp）

Seq6: GTACGAGCACACGGTGCACGTGGAGCCGGTGAGCGGCAGCCG-------------ATGTTCCTCAAGGTGAAGAAGTCGCGCTCGTAC（-13bp）

Seq7: GTACGAGCACACGGTGCACGTGGAGCCGGTGAGCGGC-------------GGCCGATGTTCCTCAAGGTGAAGAAGTCGCGCTCGTAC（-13bp）

Seq8: GTACGAGCACACGGTGCACGTGGAGCCGGTGAGCGGC-------------GGCCGATGTTCCTCAAGGTGAAGAAGTCGCGCTCGTAC（-13bp）

Seq9: GTACGAGCACACGGTGCACGTGGAGCCGGTGAGCGGCAGCCG-------------ATGTTCCTCAAGGTGAAGAAGTCGCGCTCGTAC（-13bp）

Seq10: GTACGAGCACACGGTGCACGTGGAGCCGGTGAGCGGCAGCCG-------------ATGTTCCTCAAGGTGAAGAAGTCGCGCTCGTAC（-13bp）

Seq11: GTACGAGCACACGGTGCACGTGGAGCCGGTGAGCGGCAGCCG-------------ATGTTCCTCAAGGTGAAGAAGTCGCGCTCGTAC（-13bp）

Seq12: GTACGAGCACACGGTGCACGTGGAGCCGGTGAGCGGCAGCCGTT----------------CCTCAAGGTGAAGAAGTCGCGCTCGTAC（-16bp）

Seq13: GTACGAGCACACGGTGCACGTGGAGCCGGCGAGCGGCAGCC----------------GTTCCTCAAGGTGAAGAAGCCGCGCTCGTAC（-16bp）

Seq14: GTACGAGCACACGGTGCACGTGGAGCCGGTGAGCGGCAG---------GC-------GTTCCTCAAGGTGAAGAAGTCGCGCTCGTAC（-16bp）

Seq15: GTACGAGCACACGGTGCACGTGGAGCC-----------------------------------TCAAGGTGAAGAAGTCGCGCTCGTAC（-35bp）

Seq16: GTACGAGCACACGGTGCACGTGGAGCCGGTGAGCGGCAGCCGTTACCTGCGGCCGATGTTCCTCAAGGTGAAGAAGTCGCGCTCGTAC

Seq17: GTACGAGCACACGGTGCACGTGGAGCCGGTGAGCGGCAGCCGTTACCTGCGGCCGATGTTCCTCAAGGTGAAGAAGTCGCGCTCGTAC

Seq18: GTACGAGCACACGGTGCACGTGGAGCCGGTGAGCGGCAGCCGTTACCTGCGGCCGATGTTCCTCAAGGTGAAGAAGTCGCGCTCGTAC

Seq19: GTACGAGCACACGGTGCACGTGGAGCCGGTGAGCGGCAGCCGTTACCTGCGGCCGATGTTCCTCAAGGTGAAGAAGTCGCGCTCGTAC

Seq20: GTACGAGCACACGGTGCACGTGGAGCCGGTGAGCGGCAGCCGTTACCTGCGGCCGATGTTCCTCAAGGTGAAGAAGTCGCGCTCGTAC

Seq21: GTACGAGCACACGGTGCACGTGGAGCCGGTGAGCGGCAGCCGTTACCTGCGGCCGATGTTCCTCAAGGTGAAGAAGTCGCGCTCGTAC

wnt7b No.2

Reference GTACGAGCACACGGTGCACGTGGAGCCGGTGAGCGGCAGCCGTTACATGCGGCCGATGTTCCTCAAGGTGAAGAAGTCGCGCTCGTAC

Seq1: GTACGAGCACACGGTGCACGTGGAGCCGGTGAGCGGCAGCC------TGCGGCCGATGTTCCTCAAGGTGAAGAAGTCGCGCTCGTAC（-6bp）

Seq2: GTACGAGCACACGGTGCACGTGGAGCCGGTGAGCGGCAGCCG---------GCCGATGTTCCTCAAGGTGAAGAAGTCGCGCTCGTAC（-9bp）

Seq3: GTACGAGCACACGGT---CCTCAAGGTGAAGAAGTCGCGCTCGTAC（-45bp）

Seq4: GTACGAGCACACGGTGCACGTGGAGCCG---------------------------ATGTTCCTCAAGGTGAAGAAGTCGCGCTCGTAC（-27bp）

```
Seq5:    GTACGAGCACACGGTGCACGTGGAGCCGGTGAGCGGCAGCCG-------------ATGTTCCTCAAGGTGAAGAAGTCGCGCTCGTAC（-13bp）
Seq6:    GTACGAGCACACGGTGCACGTGGAGCCGGTGAGCGGCAGCCG-------------ATGTTCCTCAAGGTGAAGAAGTCGCGCTCGTAC（-13bp）
Seq7:    GTACGAGCACACGGTGCACGTGGAGCCGGTGAGCGGCAGCCG-------------ATGTTCCTCAAGGTGAAGAAGTCGCGCTCGTAC（-13bp）
Seq8:    GTACGAGCACACGGTGCACGTGGAGCCGGTGAGCGGCAGCCG-------------ATGTTCCTCAAGGTGAAGAAGTCGCGCTCGTAC（-13bp）
Seq9:    GTACGAGCACACGGTGCACGTGGAGCCGGTGAGCGGCAGCCG-------------ATGTTCCTCAAGGTGAAGAAGTCGCGCTCGTAC（-13bp）
Seq10:   GTACGAGCACACGGTGCACGTGGAGCCGGTGAGCGGCAGCCG-------------ATGTTCCTCAAGGTGAAGAAGTCGCGCTCGTAC（-13bp）
Seq11:   GTACGAGCACACGGTGCACGTGGAGCCGGTGAGCGGC----------------CGATGTTCCTCAAGGTGAAGAAGCCGCGCTCGTAC（-16bp）
Seq12:   GTACGAGCACACGGTGCACGTGGAGCCGGTGAGCGGC----------------CGATGTTCCTCAAGGTGAAGAAGTCGCGCTCGTAC（-16bp）
Seq13:   GTACGAGCACACGGTGCACGTGGAGCCGGTGAGCGGCAGCCGTT----------------CCTCAAGGTGAAGAAGTCGCGCTCGTAC（-16bp）
Seq14:   GTACGAGCACACGGTGCACGTGGAGCCGGTGAGCGGCAGCCGTT----------------CCTCAAGGTGAAGAAGTCGCGCTCGTAC（-16bp）
Seq15:   GTACGAGCACACGGTGCACGTGGAGCCGGTGAGCGGC----------------CGATGTTCCTCAAGGTGAAGAAGTCGCGCTCGTAC（-16bp）
Seq16:   GTACGAGCACACGGTGCACGTGGAGCC--------------------TGCGGCCGATGTTCCTCAAGGTGAAGAAGTCGCGCTCGTAC（-20bp）
Seq17:   GTACGAGCACACGGTGCACGTGGAGCCGGTGAGCGGCAGCCG-------------------------GTGAAGAAGTCGCGCTCGTAC（-25bp）
Seq18:   GTACGAGCACACGGTGCACGTGGAGCCGGTGAGCGGCAGCCG-------------------------GTGAAGAAGTCGCGCTCGTAC（-25bp）
Seq19:   GTACGAGCACACGGTGCACGTGGAGCCGGTGAGCGGCAGCCG-------------------------GTGAAGAAGTCGCGCTCGTAC（-25bp）
Seq20:   GTACGAGCACACGGTGCACGTGGAGCCGGTGAGCGGCAGC----A----------------------GTGAAGAAGTCGCGCTCGTAC（-26bp）
Seq21:   GTACGAGCACACGGTGCACGTGGAGCCGGTGAG----------------------------------------AAGTCGCGCTCGTAC（-40bp）
```

Figure S9. The sequences of the target region of three genes in the lamprey larvae injected with *Cas9* mRNA and the according gRNA. **A**, The sequences of the target region of *wee1* in two injected lamprey larvae; **B**, The sequences of the target region of *soxe2* in two injected lamprey larvae; **C**, The sequences of the target region of *wnt7b* in two injected lamprey larvae. The target sites are labeled with box, the PAM sequences are highlighted with yellow and insertions are highlighted with red.

Figure S10.

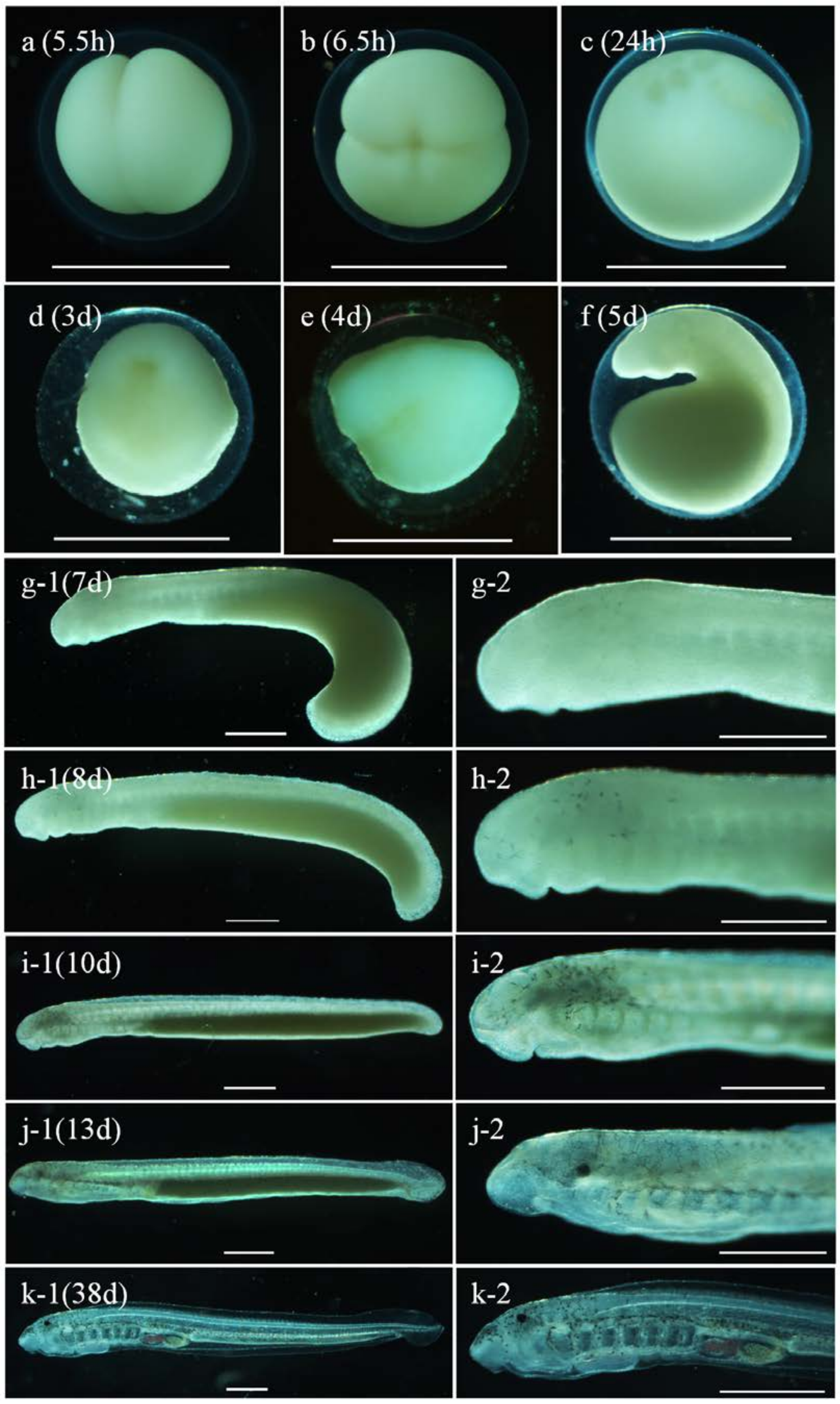

Figure S10. The development of lamprey embryos. **a**, two-cell stage, **b**, four-cell stage, **c**, blastula, **d**, gastula, **e**, neurula, **f**, lateral view of head protrusion, **g-1**, hatching, **g-2**, enlargement of g-1, **h-1**, gill pore, **h-2**, enlargement of h-1, **i-1**, eye spots, **i-2**, enlargement of i-1, **j-1**, oral hood, **j-2** enlargement of j-1. **k-1**, completion of digestive tract, **k-2**, enlargement of k-1.a, blastopore groove; b, neural fold; c, head protruded; d, melanophore; e, gill pore; f, eye spots ; g, dorsal fin; h, digestive tract. (Scale bars: 1 mm)

Table S1. The fraction of usage of each codon in the sea lamprey

Amino acid	Codon	Fraction	Amino acid	Codon	Fraction	Amino acid	Codon	Fraction	Amino acid	Codon	Fraction
Phe	TTT	0.31	Ser	TCT	0.11	Tyr	TAT	0.2	Cys	TGT	0.27
	TTC	0.69		TCC	0.22		TAC	0.8		TGC	0.73
Leu	TTA	0.03		TCA	0.1	*	UAA	-	*	UGA	-
	TTG	0.11		TCG	0.19		UAG	-	Trp	TGG	1
	CTT	0.09	Pro	CCT	0.16	His	CAT	0.23	Arg	CGT	0.11
	CTC	0.29		CCC	0.35		CAC	0.77		CGA	0.1
	CTA	0.04		CCA	0.21	Gln	CAA	0.21		CGC	0.34
	CTG	0.45		CCG	0.29		CAG	0.79		CGG	0.2
Ile	ATT	0.24	Thr	ACT	0.13	Asn	AAT	0.28	Ser	AGT	0.09
	ATC	0.64		ACC	0.33		AAC	0.72		AGC	0.3
	ATA	0.12		ACA	0.18	Lys	AAA	0.28	Arg	AGA	0.09
Met	ATG	1		ACG	0.36		AAG	0.72		AGG	0.16
Val	GTT	0.12	Ala	GCT	0.16	Asp	GAT	0.3	Gly	GGT	0.14
	GTC	0.26		GCC	0.39		GAC	0.7		GGC	0.44
	GTA	0.07		GCA	0.17	Glu	GAA	0.24		GGA	0.19
	GTG	0.55		GCG	0.29		GAG	0.76		GGG	0.23

Table S2. The target sites of five endogenous genes of lamprey

target gene	Target site*	PCR primers for amplification of the target region
wee1	CCTTGACACCTCCGCCGGGCGCC	F: CCTCACGGTGGCAAAAGTACAACGTGGG<br>R: CTTCACGGGCGACGGAGACTCCATCGACG
soxe2	GGCGGCCGTGAGTCAAGTGCTGG	F: TGTCGGATATCAATCACGAG<br>R: GGGTATTGGTCGGACAGCTT
kctd10	GGAGTCTCGCACCGAGATCGAGG	F: ACATCCAGACTCGTGTTCCTT<br>R: GAAATACGCGATGATACCTGC
wnt7b	CCGTTACCTGCGGCCGATGTTCC	F: TTATCGCCGTTTCCACTCCA<br>R: TACTGGTGCGTGTTGTAGCC
golden	GGGTCAACGACTTCCTGCACGGG	F: CTCTCGCTCGTGTCCGTCT<br>R: CCGTGCAGGCTGAGCGTCAACTGCG

* The sequence underlined is the PAM region. The sequence highlighted is the sequence required for T7 RNA polymerase starting site

Table S3. The ratio of the larvae with hypopigmentation in the retinas when injected with Cas9 and *gol* gRNA in zebrafish

injection	Total injected larvae	Normal pigment	No pigment	Mosaic pigment	Mutants
control	100	100	0 (0%)	0 (0%)	0
ZCas9 + *gol* gRNA	46	4	4 (8.7%)	38 (82.6%)	91.3%
